# Hippocampal hub neurons maintain distinct connectivity throughout their lifetime

**DOI:** 10.1038/s41467-020-18432-6

**Published:** 2020-09-11

**Authors:** Marco Bocchio, Claire Gouny, David Angulo-Garcia, Tom Toulat, Thomas Tressard, Eleonora Quiroli, Agnès Baude, Rosa Cossart

**Affiliations:** 1 INMED (INSERM U1249), Aix-Marseille University, Turing Center for Living Systems (CENTURI), Parc Scientifique de Luminy, 13273 Marseille, France; 2 Present Address: Grupo de Modelado Computacional–Dinámica y Complejidad de Sistemas, Instituto de Matemáticas Aplicadas, Universidad de Cartagena, 130001 Cartagena, Colombia

**Keywords:** Cellular neuroscience, Development of the nervous system, Neural circuits, Neuronal physiology

## Abstract

The temporal embryonic origins of cortical GABA neurons are critical for their specialization. In the neonatal hippocampus, GABA cells born the earliest (ebGABAs) operate as ‘hubs’ by orchestrating population synchrony. However, their adult fate remains largely unknown. To fill this gap, we have examined CA1 ebGABAs using a combination of electrophysiology, neurochemical analysis, optogenetic connectivity mapping as well as ex vivo and in vivo calcium imaging. We show that CA1 ebGABAs not only operate as hubs during development, but also maintain distinct morpho-physiological and connectivity profiles, including a bias for long-range targets and local excitatory inputs. In vivo, ebGABAs are activated during locomotion, correlate with CA1 cell assemblies and display high functional connectivity. Hence, ebGABAs are specified from birth to ensure unique functions throughout their lifetime. In the adult brain, this may take the form of a long-range hub role through the coordination of cell assemblies across distant regions.

## Abstract (web summary)

In the neonatal hippocampus, GABA cells born the earliest operate as ‘hubs’ by orchestrating population synchrony. Here, the authors show that the earliest born GABAergic cells in the hippocampal CA1 region maintain distinct anatomical and functional properties throughout their lifetime.

## Introduction

GABAergic neurons are a critical component of cortical circuit development and function. This sparse and heterogeneous population of cells has been classified according to several parameters, including genetic and molecular markers, connectivity schemes as well as morphological and electrophysiological properties^1^. According to leading theories, this heterogeneity greatly enhances the computational power of cortical networks^2^.

The diversity among GABAergic cells is specified as early as progenitor stages in the ganglionic eminences, the embryonic regions that give rise to cortical inhibitory neurons^3,4^. Genetic restriction of neuronal potential from spatially distributed progenitors is a major determinant of GABA neuron diversity^5–7^. In addition, a temporal clock also shapes GABA neuron fate: discrete temporal windows within the same ganglionic eminence specify different cell types^8,9^. Hence, place and time of origin from discrete progenitor pools in the ganglionic eminences determine, at least in part, the ultimate features that the cell will display in adult cortical circuits.

The temporal control of GABA neurons’ fate is particularly striking when considering early born cells. In the CA3 region of the hippocampus, early born GABAergic neurons (ebGABAs) act as “hubs” during the perinatal period, that is they show exceptional functional and effective connectivity^10^. Due to this remarkable connectivity scheme, CA3 hub cells anticipate and coordinate single-handedly spontaneous network bursts occurring in the form of giant depolarizing potentials (GDPs) in postnatal slices^10,11^. Interestingly, these cells are still present in adult animals and a fraction of them display long-range projections to the medial septum^12^.

It is not known whether ebGABAs are able to orchestrate network synchrony only in CA3, a region that is characterized by notable recurrent connectivity and that is the preferred site of GDP initiation^13^. In addition, despite their major developmental role, the integration and function of ebGABAs into adult circuits remains unknown. More generally, it is not known whether an early neuronal birth date leads to distinct functional properties in adult circuits. To fill this gap, we have examined ebGABAs in the CA1 region of the hippocampus, because this area is well understood in terms of cell types and connectivity^14^ and is more accessible for in vivo calcium imaging. We investigated ebGABAs’ morphological and electrophysiological properties, their local and long-range connectivity as well as their involvement in network dynamics. We show that CA1 ebGABAs operate as hub cells during the early postnatal period and maintain distinct properties in adulthood, encompassing neurochemical content, intrinsic electrophysiological properties, input connectivity and in vivo activity.

## Results

### CA1 ebGABAs are sparse and mostly located in deep layers

To study CA1 ebGABAs, we used the inducible transgenic driver line Dlx1/2-Cre^ER^, expressing Cre^ER^ under the control of the Dlx1/2 intergenic enhancer^15^. We crossed Dlx1/2-Cre^ER^ mice with a floxed GFP reporter line (see Methods). Like in our previous studies^10,12^, ebGABAs were labeled with GFP by inducing Cre recombinase at embryonic day 7.5 or 8.5 (E7.5 or E8.5) via tamoxifen administration. For simplicity, we refer to this tamoxifen-treated transgenic line as Dlx1/2(E7.5)-GFP. Since *Dlx1* and/or *Dlx2* genes are required for the proper development of all GABAergic cells^16^, this approach labels GABAergic neurons from all ganglionic eminences. However, it is likely to label more medial ganglionic eminence (MGE)-derived neurons, which on average are born earlier than caudal ganglionic eminence (CGE)-derived cells^6^.

**Fig. 1:**
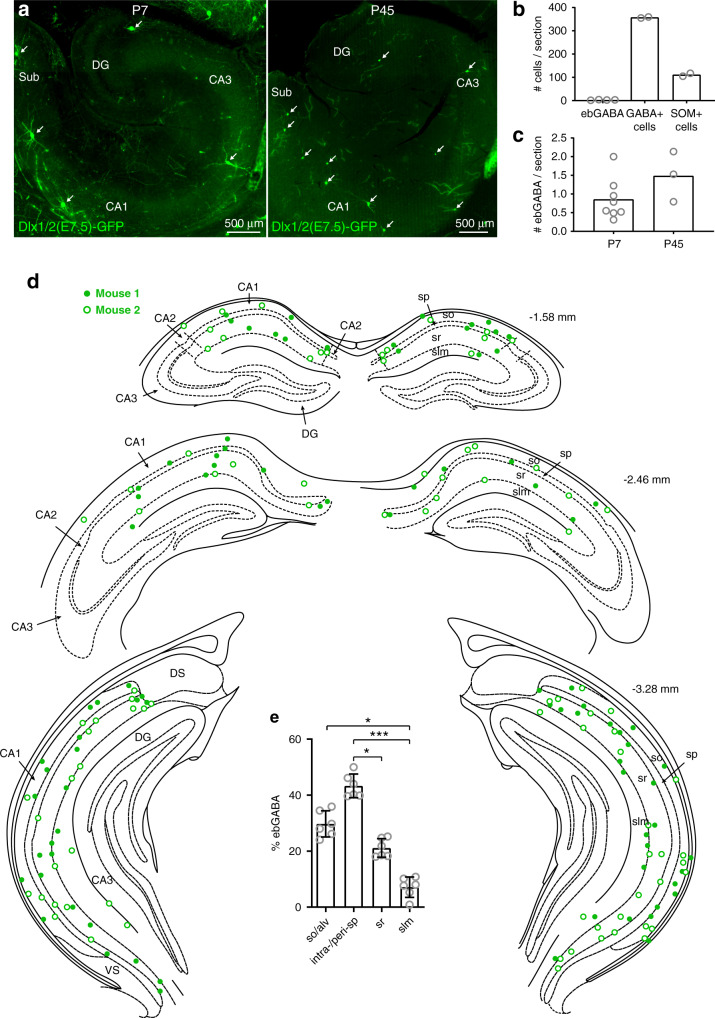
Distribution of ebGABAs in the CA1 region of the hippocampus. **a** Epifluorescence images showing sparse ebGABAs’ somata (GFP+ cells, arrows) in horizontal sections containing the hippocampus of P7 (left*)* and P45 (right) Dlx1/2(E7.5)-GFP mice. DG dentate gyrus, Sub subiculum. This experiment was repeated independently in seven mice for P7 and in three mice for P45, obtaining similar results. **b** Number of ebGABAs (170 cells from 4 brains), GABA+ cells (719 cells from 2 brains) and SOM+ cells (533 cells from 2 brains) in the whole hippocampus of P7 mice per 70 μm-thick coronal section. **c** Number of CA1 ebGABAs per 80 μm-thick horizontal section at P7 (116 cells from 7 brains) and at P45 (135 cells from 3 brains). **d** Position of CA1 ebGABAs mapped in two different brains from Dlx1/2(E7.5)-GFP mice at three different rostrocaudal coordinates at P60. At each rostrocaudal level, ebGABAs were mapped by collapsing three neighboring 70 µm-thick coronal sections. EbGABAs with somata in CA2, CA3, dentate gyrus (DG), dorsal subiculum (DS), and ventral subiculum (VS) were omitted for clarity. **e** Significant effect of layering in the proportional distribution of CA1 ebGABAs (*P* < 0.0001, *n* = 6 brains, Friedman test; so vs. intra-/peri-sp *P* > 0.99, so vs. sr *P* > 0.99, so vs. slm *P* = 0.0439, intra-/peri-sp vs. sr *P* = 0.0439, intra-/peri-sp vs. slm *P* = 0.0003, sr vs. slm *P* > 0.99, two-sided Dunn’s multiple comparisons test). Bar plots represent means. Error bars represent SDs. so stratum oriens, sp stratum pyramidale, sr stratum radiatum, slm stratum lacunosum-moleculare, *P < 0.05; **P < 0.01; ***P < 0.001. Source data are provided as a Source Data file.

In line with our previous reports^10,12^, Dlx1/2(E7.5)-GFP ebGABAs of the hippocampus were very sparse (3 ± 1 cells per 70 μm-thick PFA-fixed coronal section at P7, mean ± SD, 58 sections from four mice, quantified bilaterally, Fig. 1a–c). We estimated that the amount of ebGABAs labeled with our approach is ~1% of GABA-positive cells and is ~20 times lower than the amount of somatostatin-positive (SOM+) cells (Fig. 1b). In CA1, ebGABAs were similarly sparse at neonatal and adult stages: 0.8 ± 0.5 ebGABAs per 80 μm-thick horizontal section at P7 (171 sections from 7 mice), 1.5 ± 0.6 ebGABAs per section at P45 (77 sections from 3 mice, quantified bilaterally, Fig. 1c). Next, we examined the distribution of ebGABAs’ somata in the rostrocaudal and dorsoventral axes (*n* = 2 P45 mice, Fig. 1d), observing that these cells were sparse and scattered in a relatively even fashion across the entire CA1 region.

Next, we studied their distribution across CA1 layers (six P45 mice). The location of ebGABAs’ somata appeared to be significantly dependent on layering (*P* < 0.0001, Friedman test, Fig. 1e). The somata of most ebGABAs were located in the stratum oriens and inside or around the stratum pyramidale (29 ± 5% and 43 ± 4%, respectively, means ± SDs). Fewer cells (21 ± 4%) were located in the stratum radiatum and only a small minority (7 ± 4%) populated the stratum lacunosum-moleculare.

### EbGABAs are operational hub cells in the developing CA1

We sought to test whether ebGABAs played a hub function in the developing CA1 circuit. To this end, horizontal slices (380 µm-thick) from Dlx1/2(E7.5)-GFP or littermate GFP-negative controls containing the intermediate/ventral CA1 were loaded with the calcium indicator Fura2-AM. Slices were subsequently imaged using two-photon microscopy to record spontaneous neuronal activity (Supplementary Movie 1). EbGABAs were generally very sparse (1.5 ± 1.1 cells found per hippocampus, quantified unilaterally from four mice, mean ± standard deviation, SD). Three reasons are likely to contribute to the fact that the number of ebGABAs was lower in acute experiments than in histological analyses from fixed tissue. First, the signal from fixed slices was amplified with immunofluorescence; second, some ebGABAs may not survive after the slicing procedure; third, the GFP signal could not be detected below ~100 µm in depth in acute slices. As we previously reported for CA3 ebGABAs^10^, GFP+ cells could not be labeled with Fura2-AM. This prevented us from calculating their functional connectivity index based on the analysis of spontaneous calcium events. Thus, we tested the role of ebGABAs in orchestrating spontaneous network dynamics by measuring the effect of ebGABA stimulation on GDPs.

**Fig. 2:**
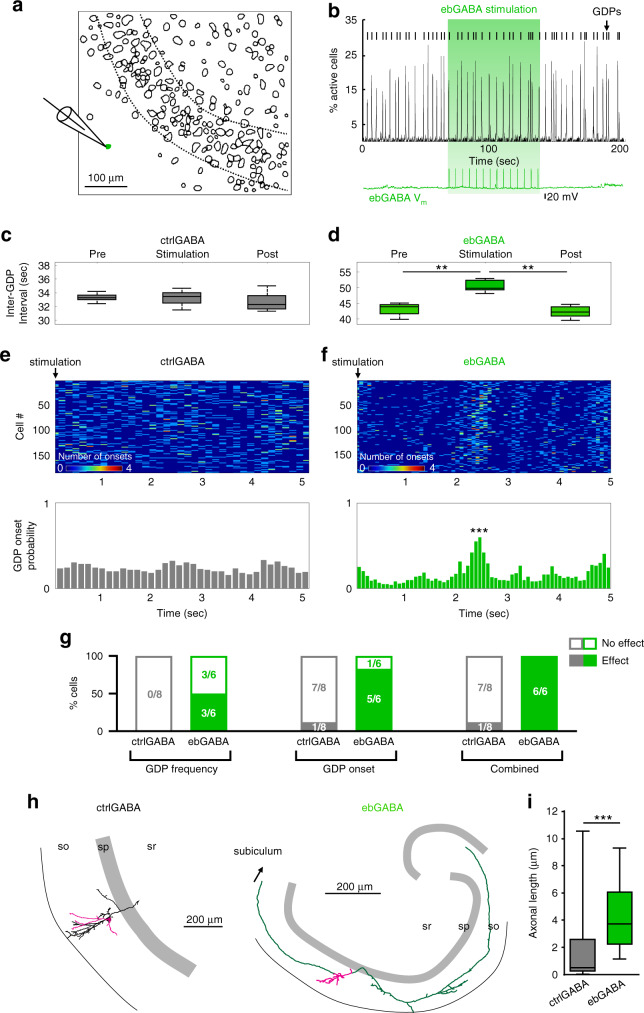
ebGABAs orchestrate network activity in the developing CA1. **a** Detected contours of imaged CA1 cells. An ebGABA (green cell) was patched and stimulated by injecting suprathreshold depolarizing current steps. Dashed lines delimit the stratum pyramidale. **b** Histogram displaying the percentage of active cells in the field of view. **c**, **d** Box plots of “Inter GDP intervals” of a representatives ctrlGABA and ebGABA cell. **c** stimulation of the ctrlGABA cell does not affect the inter GDP interval (*P* > 0.1, two-sided Mann–Whitney U test, *n* = 14 trials). **d** stimulation of the ebGABA cell increases the inter GDP interval (*P* < 0.01, Mann–Whitney *U* test, *n* = 14 trials). Data are represented as medians and interquartile ranges. Error bars represent minimum and maximum values. **e** Stimulation of the ctrlGABA does not synchronize GDP occurrence. Top, raster plot of calcium onsets following each stimulation (each row represents the average activity of single cell across 20 trials). Bottom, histogram showing no significantly increase in the probability of calcium events following ctrlGABA stimulation. **f** Stimulation of representative ebGABA synchronizes GDP occurrence. Top, raster plot of calcium transient onsets following each stimulation (each row represents the average activity of a cell across 20 trials). Colorbar depicts number of onsets. Bottom, histogram showing significant increase in the GDP probability following ebGABA stimulation (*P* < 0.001, permutation test). **g** Proportions of ctrlGABA and ebGABA displaying significant effects on GDP frequency and GDP synchronization upon stimulation. A significantly higher proportion of ebGABAs impacts GDP frequency and/or synchronization (right, *P* = 0.0014, Fisher’s exact test). **h** Neurolucida reconstructions of representative neurobiotin-filled ctrlGABA and ebGABA cells. Axon is depicted in green for ebGABA and in black for ctrlGABA. Soma and dendrites are colored in magenta. **i** Boxplot comparing axonal lengths obtained in Neurolucida reconstructed ctrlGABA (*n* = 20) and ebGABA cells (*n* = 18). EbGABA display significantly higher axonal length than ctrlGABA (*P* = 0.0006, two-sided Mann–Whitney *U* test). Panels **b** and **d** represent the same ebGABA cell. All the other panels represent different cells. Boxplots represent medians (center), interquartile ranges (bounds), minima and maxima (whiskers). **P < 0.01, ***P < 0.001. so stratum oriens, sp stratum pyramidale, sr stratum radiatum. Source data are provided as a Source Data file.

We performed whole cell patch clamp recordings from ebGABA (*n* = 65) and ctrlGABA cells (random putative GABAergic cells in stratum oriens and stratum radiatum, *n* = 17, Fig. 2a, b). A phasic stimulation protocol was applied, i.e., short supra-threshold current pulses repeated at 0.1, 0.2, and 0.4 Hz (within the frequency range of spontaneous GDP occurrence). GDPs could be detected only in 14/82 slices (including 8 ctrlGABAs and 6 ebGABAs). Thus, subsequent analyses were restricted to these cases. Stimulation of 3/6 ebGABAs significantly affected GDP frequency (among these, 2 decreased GDP frequency whereas one increased it, Fig. 2d, g). In contrast, no stimulated ctrlGABA (0/8) significantly affected GDP frequency (Fig. 2c, e and Supplementary Table 1). This difference was unlikely to arise from spontaneous differences in GDP rate between Cre^ER^-positive and Cre^ER^-negative mice because the median GDP intervals of ctrlGABA and ebGABA experiments were comparable (ctrlGABA: median 0.02 Hz, interquartile ranges (IQR): 0.014–0.022 Hz; ebGABA: median = 0.017, IQR: 0.015–0.023, *P* > 0.99, Mann–Whitney *U* test).

Next, we asked whether ebGABAs could also synchronize the network in the shorter timescale. We examined the occurrence of calcium events in all the trials that followed the depolarizing current steps used as stimulation. We constructed a calcium event probability histogram including the activity of all the cells in the field of view and we normalized by the number of GDPs during the stimulation protocol. Within interstimulations trials, ebGABA activation increased synchronous calcium activity (Fig. 2f).

To define cells that significantly locked the onset of GDPs, we examined whether the highest peak of the calcium event probability histogram passed a threshold defined using a surrogate distribution (see “Methods” for details). Using this method, we calculated that 6/6 ebGABAs significantly synchronized the onset of GDPs, with GDP onset occurring 2.2 ± 0.9 s after ebGABA activation (mean ± SD). In contrast, only one ctrlGABA was found to significantly lock GDP onset (Fig. 2e–g and Supplementary Table 1).

Overall, 1/6 ebGABA increased the frequency of GDPs and synchronized GDP occurrence, 2/6 ebGABAs decreased the frequency of GDPs and synchronized GDP occurrence, and 3/6 ebGABAs synchronized GDP occurrence but had no effect on GDP frequency. In contrast, only 1/8 ctrlGABA synchronized GDP occurrence. The remaining ctrlGABAs had no effect on either GDP frequency or onset (Supplementary Table 1). On balance, all ebGABAs could affect the coordination of neuronal activity in the developing CA1 network, whereas the proportion of ctrlGABA affecting network dynamics (1/8) was significantly lower (*P* = 0.0014, Fisher’s exact test). Hence, CA1 ebGABAs can be classified as operational hub neurons because they can synchronize the activity of large population of cells in the network.

### EbGABAs show remarkably long axons in the developing CA1

Previously described hub cells and ebGABAs in the CA3 area could be distinguished by four times longer axonal lengths compared to non-hub cells^10,11^. We asked whether widespread axonal arborizations could also be a distinctive feature of CA1 ebGABAs. To test this, we reconstructed 38 neurobiotin-filled neurons (ctrlGABAs: *n* = 20, ebGABAs: *n* = 18, Fig. 2h and Supplementary Fig. 1).

The axons of most CA1 ebGABAs displayed remarkable lengths, in some cases crossing CA1 boundaries and innervating CA3 and/or the subiculum (Fig. 2h and Supplementary Fig. 1). In four cases, the axons ran in the alveus, suggesting a possible extrahippocampal projection. We performed morphometric analysis on the reconstructed cells. In line with previous results on CA3 ebGABA and hub cells, CA1 ebGABAs showed significantly longer axons (*P* = 0.0006) that covered a significantly larger surface than ctrlGABAs (*P* = 0.0003, Mann–Whitney U test, Fig. 2i and Supplementary Fig. 2a). In contrast, dendritic length or surface covered by dendrites did not differ significantly between the two groups (*P* = 0.279 and *P* = 0.125, respectively, Mann–Whitney *U* test, Supplementary Fig. 2b). When we pooled cells that had a significant effect on GDPs (operational hub cells, six ebGABAs and one ctrlGABA), we found that hub cells had significantly longer axons (but not dendrites) than non-hub cells (seven ctrlGABAs, *P* = 0.0379, Mann–Whitney *U* test, Supplementary Fig. 2c, d), pointing toward a link between widespread axons and an operational hub role. Thus, CA1 ebGABAs exhibit functional and anatomical features of previously reported hub cells^10,11,17^.

### Adult ebGABAs exhibit features of long-range projecting cells

**Fig. 3:**
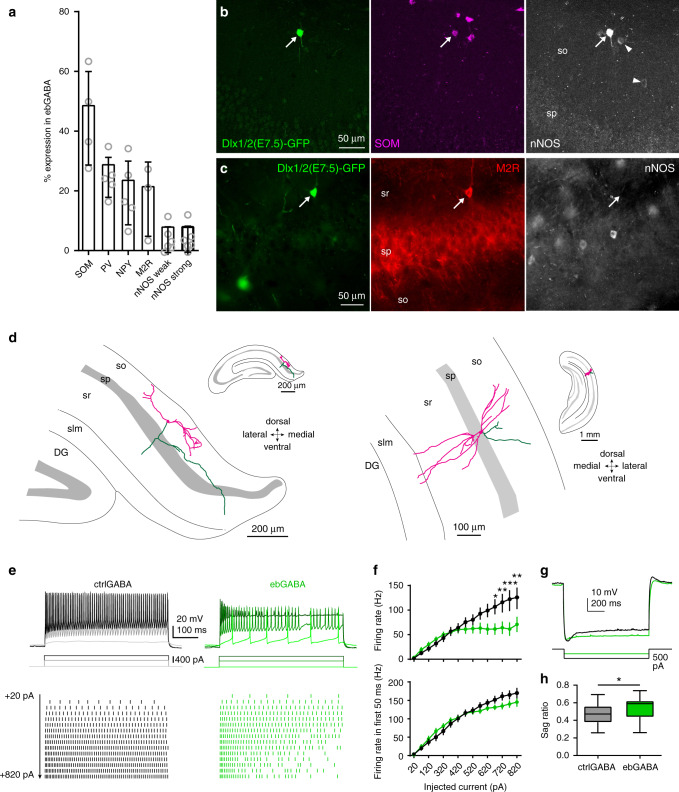
Adult ebGABAs display distinct anatomical and electrophysiological properties. **a** Quantification of the expression of single molecular markers in CA1 ebGABA. SOM: *n* = 4 mice; PV: *n* = 5 mice; NPY: *n* = 5 mice; M2R: *n* = 3 mice; nNOS weak/strong: *n* = 5 mice. Bar plots represent means. Error bars represent SDs. **b** Example ebGABA in stratum oriens expressing SOM and strong levels of nNOS (arrow). Note that neurons expressing weak levels of nNOS (arrowheads) are SOM immunonegative. Experiment repeated independently in four mice with similar results. **c** EbGABA in stratum radiatum expressing only M2R but not nNOS (arrow). Experiment repeated independently in three mice, obtaining similar results. **d** Neurolucida reconstructions of representative neurobiotin-filled ebGABA in the adult CA1. Axon is depicted in green. Soma and dendrites are colored in magenta. Inset: zoomed out representation to illustrate the rostro-caudal and dorso-ventral position. **e** Representative responses to +20, +520, and +820 pA current injections in ctrlGABA and ebGABA. Note the lower firing rate of the ebGABA in response to stronger current injections. **f** Top, f/I plot showing reduced excitability of ebGABA with strong current injections. Effect of current injection *F*(14,864) = 31.59, *P* < 0.0001; effect of birth date *F*(7,864) = 16.40, *P* < 0.0001, interaction *F*(98, 864) = 1.881 *P* < 0.0001, two-way ANOVA with Bonferroni post hoc test. Bottom, f/I plot limited to the first 50 ms of the current injection. Effect of current injection *F*(14,461) = 77.8, *P* < 0.0001; effect of birth date *F*(1,461) = 3.211, *P* = 0.0738, interaction *F*(1,461) = 1.676, *P* = 0.0573, two-way ANOVA with Bonferroni multiple comparisons test. CtrlGABAs: *n* = 18; ebGABAs: *n* = 16. Data are represented as means ± standard errors of the means. **g** Representative sag response of a ctrlGABA (black trace, −500 pA current injection) and an ebGABA (green trace, −250 pA current injection). Each trace is an average of three sweeps. **h** EbGABAs (*n* = 16 cells) display a significantly higher sag ratio (i.e., a lower sag amplitude) than ctrlGABAs (*n* = 18 cells, *P* = 0.028, two-sided Mann–Whitney *U* test). Boxplots represent medians (center), interquartile ranges (bounds), minima and maxima (whiskers). *P < 0.05; **P < 0.01; ***P < 0.001. so stratum oriens, sp stratum pyramidale, sr stratum radiatum, slm stratum lacunosum-moleculare. Source data are provided as a Source Data file.

Given that ebGABAs displayed unique anatomical and functional features in the immature CA1, we asked whether they maintained distinct properties in adulthood. We examined the molecular content of CA1 ebGABAs to infer the putative cell types comprising this GABAergic population. Staining for single neurochemical markers, we found that many ebGABAs expressed SOM (49 ± 16%, mean ± SD, four mice) and, in a progressively lower extent, PV (29 ± 7%, five mice), NPY (24 ± 11%, five mice) and M2R (22 ± 12%, three mice, Fig. 3a and Supplementary Fig. 3a, b). These data are in line with previously published results on the whole hippocampus^10^. Using an antibody that allows discrimination between weak and strong levels of nNOS expression, we found that a small but consistent proportion of ebGABAs (8 ± 4%, six mice) expressed strong nNOS levels, a marker of long-range projection cells^18^ (Fig. 3b and Supplementary Fig. 3c).

To estimate the cell types comprised in the ebGABA population, we examined the combinatorial expression of molecular markers in these cells. In the hippocampus, strong nNOS+ cells are GABAergic projection cells that also express SOM, NPY and M2R^14^ and are likely to innervate the dentate gyrus and CA3^19^.

We confirmed that strong nNOS+ ebGABAs belong to this cell type by finding that these cells often co-expressed SOM (94 ± 11%, four mice), NPY (93 ± 13%, five mice) and M2R (62 ± 27%, three mice, Fig. 3b and Supplementary Fig. 3). EbGABAs in stratum oriens often expressed SOM and NPY but rarely PV, confirming that the majority of ebGABAs in this layer are not O-LM cells. Furthermore, some ebGABA (15 ± 7%) expressed M2R but not nNOS (three mice, Fig. 3c), suggesting that some of these cells could be retrohippocampal projection cells innervating the subiculum and the retrosplenial cortex^20^.

A recent study pointed toward COUP-TFII as a possible temporal identity cue promoting an early specification toward SOM+ GABA neurons^21^. Since most ebGABAs are SOM+, we asked whether COUP-TFII could be a broad ebGABA marker. However, only 7 ± 2% of ebGABAs were COUP-TFII+ (five mice, Supplementary Fig. 3i). Based on the combinatorial expression of molecular markers and previous data^10,12^, we estimated that at least 40% of ebGABA are likely to be long-range GABAergic projection cells (see “Methods” for details). As it was estimated that CA1 GABAergic projection cells account for only 5–7% of all GABAergic cells^22^, these data suggest that ebGABAs are biased toward a long-range output connectivity.

To corroborate these data, we filled ebGABAs with neurobiotin using whole cell patch clamp in acute coronal brain slices (*n* = 34 cells). A subset of these cells (*n* = 14) were morphologically reconstructed to reveal their axonal and dendritic arborizations (Fig. 3d and Supplementary Fig. 4). In line with the immunohistological data, all reconstructed ebGABAs displayed little local axonal arborizations, suggesting that most of these neurons do not belong to canonical interneuron types. In addition, the axons of some ebGABAs consisted of only one or two branches running straight for several hundreds of micrometers. Finally, not only the somata of ebGABAs were rarely located in stratum lacunosum-moleculare, but also ebGABAs’ dendrites arborized very little in this layer. Taken together, these data indicate that ebGABAs are biased toward a deep radial location and long-range outputs.

### Adult ebGABAs have distinct electrophysiological properties

We then wished to determine whether ebGABAs mature into an electrophysiologically defined subpopulation of GABA neurons. To this end, we performed a series of ex vivo whole cell patch clamp recordings in acute brain slices from adult Dlx1/2(E7.5)-GFP mice (*n* = 167 cells from 73 mice; 85 ctrlGABAs, 82 ebGABAs), sampling from both the dorsal and intermediate/ventral CA1. The scarcity of ebGABAs in acute slice experiments from adult mice was similar to acute slice experiments at neonatal stage (1.6 ± 0.8 cells found per hippocampus, quantified unilaterally from five mice, mean ± SD). First, we analyzed ebGABAs intrinsic electrophysiological properties (ctrlGABAs: *n* = 18; ebGABAs: *n* = 16). Consistent with the fact that many ebGABAs express SOM+, these cells displayed relatively long spikes (half-width: 0.9 ± 0.3 ms, mean ± SD) and a regular, non-fast-spiking pattern of discharge (firing rate at 2× rheobase: 24 ± 17 Hz; adaptation index: 0.69 ± 0.09, Supplementary Table 2).

Analysis of firing rate vs. injected current (f/I) curves revealed a sublinear input/output relationship in ebGABA but not in ctrlGABA (Fig. 3f, g; effect of current injection *P* < 0.0001; effect of birth date *P* < 0.0001, interaction *P* < 0.0001, two-way ANOVA). In many cases, upon strong current injections ebGABA displayed initial high frequency firing, but then inactivated, likely due to depolarization block^23^. When we plotted an f/I curve only for a shorter time window of the current injection (the initial 50 ms), the firing of ebGABA was not significantly lower than the one of ctrlGABA (effect of current injection *P* < 0.0001; effect of birth date *P* = 0.0738). In addition, ebGABAs showed a significantly smaller “sag” response (i.e., a significantly higher sag ratio, *P* = 0.028, Mann–Whitney *U* test, Fig. 3g, h). The remaining intrinsic membrane parameters that we examined did not differ between the two groups (Supplementary Table 2). These data suggest that an early birth date biases GABAergic cells toward specific electrophysiological properties.

### EbGABAs are mostly excited by intra-hippocampal inputs

Since the somata of ebGABAs are preferentially located in deep CA1 layers and CA1 inputs are radially organized, we asked whether an early birth date biases GABAergic cells toward a special input connectivity from glutamatergic afferents. We probed ebGABAs’ monosynaptic input connectivity from specific pathways using electrical stimulation and optogenetic mapping combined with intracellular voltage clamp recordings (with cells held at *E*_Cl_: −87 mV).

**Fig. 4:**
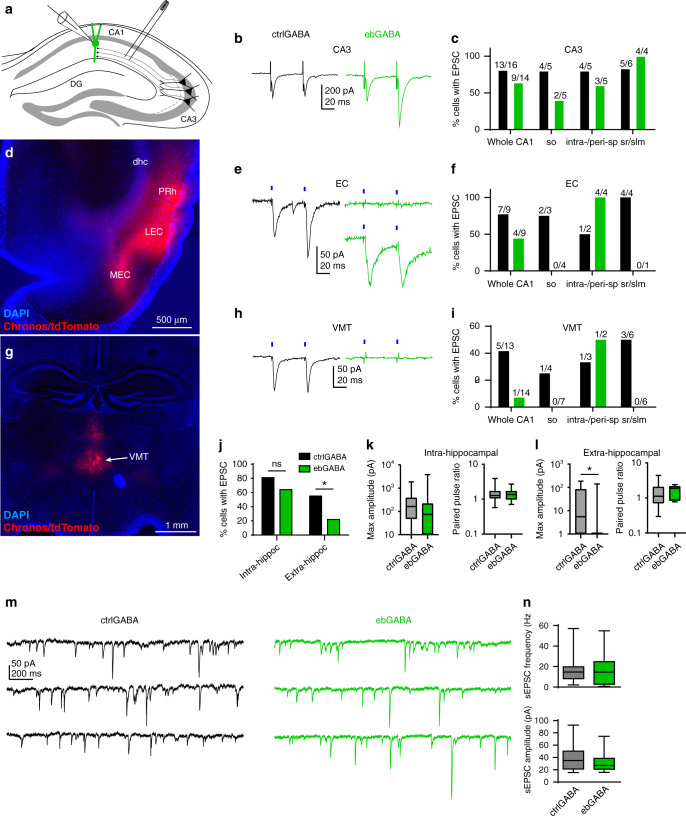
Sparse extra-hippocampal excitatory input connectivity onto ebGABAs. **a** Schematic of the experimental configuration: electrical stimulation of the Schaffer collateral. **b** CtrlGABA and ebGABA responding to CA3 stimulation. Stimulation artifacts were trimmed for clarity. **c** Proportions of ctrlGABAs and ebGABAs responding to CA3 stimulation. **d** Representative injection site for EC stimulation experiments. Experiment repeated in 15 mice with similar results. **e** CtrlGABA responding to EC stimulation and two ebGABAs: one responsive and one nonresponsive. **f** Proportions of ctrlGABAs and ebGABAs responding to EC stimulation. **g** Representative injection site for VMT stimulation experiments. Experiment repeated in seven mice with similar results. **h** CtrlGABA responding to VMT stimulation and ebGABA displaying no detectable response. **i** Proportions of ctrlGABAs and ebGABAs responding to VMT stimulation. **j** The proportions of ctrlGABAs and ebGABAs receiving intra-hippocampal excitatory inputs (CA3) were not significantly different (*P* = 0.417, two-sided Fisher’s exact test, *n* = 16 ctrlGABAs, *n* = 14 ebGABAs). The proportion of ebGABA receiving extra-hippocampal excitatory inputs (pooled EC and VMT data) was significantly lower than the proportion of ctrlGABAs (*P* = 0.0331, two-sided Fisher’s exact test, *n* = 22 ctrlGABA, *n* = 23 ebGABA). **k** No significant difference in maximum EPSC amplitude (*P* = 0.29, two-sided Mann–Whitney *U* test, *n* = 16 ctrlGABAs, *n* = 14 ebGABAs) or paired pulse ratio (*P* > 0.99, two-sided Mann–Whitney *U* test, *n* = 13 ctrlGABAs, *n* = 9 ebGABAs) for intra-hippocampal afferents. **l** Significant difference in maximum EPSC amplitude (*P* = 0.024, two-sided Mann–Whitney *U* test, *n* = 22 ctrlGABAs, *n* = 23 ebGABAs) but not paired pulse ratio (*P* = 0.72, two-sided Mann–Whitney *U* test, *n* = 11 ctrlGABAs, *n* = 5 ebGABAs) for extra-hippocampal afferent stimulation between ctrlGABAs and ebGABAs. **m** Example sEPSCs recorded from ctrlGABA and ebGABA cells. **n** Similar sEPSC frequency (*P* = 0.4, Mann–Whitney *U* test) and amplitude (*P* = 0.42, two-sided Mann–Whitney *U* test) in ctrlGABAs (*n* = 18 cells) and ebGABAs (*n* = 17 cells). *x*-axis in **c**, **f**, and **i** represent somatic location of the recorded neurons. Boxplots represent medians (center), interquartile ranges (bounds), minima and maxima (whiskers). *P < 0.05. LEC lateral entorhinal cortex, MEC medial entorhinal cortex, PRh perirhinal cortex, dhc dorsal hippocampal commissure, VMT ventromedial thalamus, so stratum oriens, sp stratum pyramidale, sr stratum radiatum, slm stratum lacunosum-moleculare. Source data are provided as a Source Data file.

First, we focused on the Schaffer collateral input by stimulating the axons from CA3 pyramidal cells in stratum radiatum using a bipolar stimulating electrode while recording from CA1 GABA cells in voltage clamp (Fig. 4a). Electrical stimulation of the Schaffer collateral evoked EPSCs in the majority of ctrlGABAs (13/16 cells) and ebGABAs (9/14 cells, Fig. 4b, c). EPSCs were mediated by AMPA/KA receptors because they were blocked by NBQX (10 µM, 3/3 cells, Supplementary Fig. 5). Thus, the majority of ebGABAs are recruited by CA3 inputs.

Since somata and dendrites of ebGABAs are rarely found in the stratum lacunosum-moleculare, we predicted that long-range inputs targeting this layer would play a less important role in the recruitment of these cells. To evaluate this hypothesis, we tested the inputs from the entorhinal cortex (EC) and the ventromedial thalamus (VMT) using a viral vector to express the fast opsin Chronos in these regions (Fig. 4d–i). Following at least two weeks of expression, Chronos/tdTomato+ axons densely innervated the stratum lacunosum-moleculare of CA1 for both injection sites (some axons were also detected in the stratum oriens next to the alveus, Supplementary Fig. 5; very few axons were also present in other layers). Pulses of 470 nm light were delivered to test for the presence of short-delay EPSCs (monosynaptic connections) in the recorded cells. For EC stimulations, we detected EPSCs in the majority of ctrlGABAs (7/9 cells), but only in about half of the ebGABAs (4/9 cells, Fig. 4e, f). For VMT stimulations, we detected EPSCs in 5/13 ctrlGABAs cells but only in 1/14 ebGABAs (Fig. 4h, i).

The proportion of ebGABA recruited by an intra-hippocampal excitatory input (CA3) was not significantly different from the proportion of ctrlGABA (*P* = 0.417, Fisher’s exact test). In contrast, the proportion of ebGABA recruited by extra-hippocampal excitatory inputs (EC and VMT pooled) was significantly lower than the proportion of ctrlGABA (5/23 and 12/22, respectively, *P* = 0.0331, Fisher’s exact test, Fig. 4j). In line with this, maximum EPSC amplitude for intra-hippocampal afferents did not differ between the two populations (*P* = 0.29, Mann–Whitney *U* test, *n* = 16 ctrlGABAs, *n* = 14 ebGABAs), whereas maximum amplitude for extra-hippocampal afferents was lower for ebGABAs (*P* = 0.024, Mann–Whitney *U* test, *n* = 22 ctrlGABAs, *n* = 23 ebGABAs, Fig. 4k, l). However, paired pulse ratios for intra- and extra-hippocampal afferents were similar in the two populations (*P* > 0.99, *n* = 13 ctrlGABAs, *n* = 9 ebGABAs and *P* = 0.72, *n* = 11 ctrlGABAs, *n* = 5 ebGABAs, respectively, Mann–Whitney U test). Maximum EPSC amplitudes for extra-hippocampal afferents were comparable when only responsive cells were taken into account (Supplementary Fig. 5f, g). This indicates that an early birth date biases GABA cells’ input connectivity but not synaptic strength or release probability. Given the sparse connectivity and the fact that EPSCs evoked in ebGABAs by stimulation of the EC were small (maximum amplitude 19 ± 33 pA, mean ± SD), these data suggest that extra-hippocampal afferents play a small role in the recruitment of ebGABAs.

Furthermore, we examined spontaneous EPSCs (sEPSCs), many of which are likely to be action potential-dependent, and thereby arise from intra-hippocampal connections in coronal slices. We confirmed that sEPSCs were mediated by AMPA/KA receptors because they were completely blocked by NBQX (10–20 µM, 4/4 cells). CtrlGABA and ebGABA displayed similar sEPSC parameters, in particular frequency and amplitude (Fig. 4k, l and Supplementary Fig. 5h), indicating similar recruitment of these cells by putative intra-hippocampal excitatory afferents. Taken together, these data indicate that the deep location and dendritic arborization of CA1 ebGABA favor their recruitment by intra-hippocampal inputs.

### ebGABAs receive weak local synaptic inhibition

**Fig. 5:**
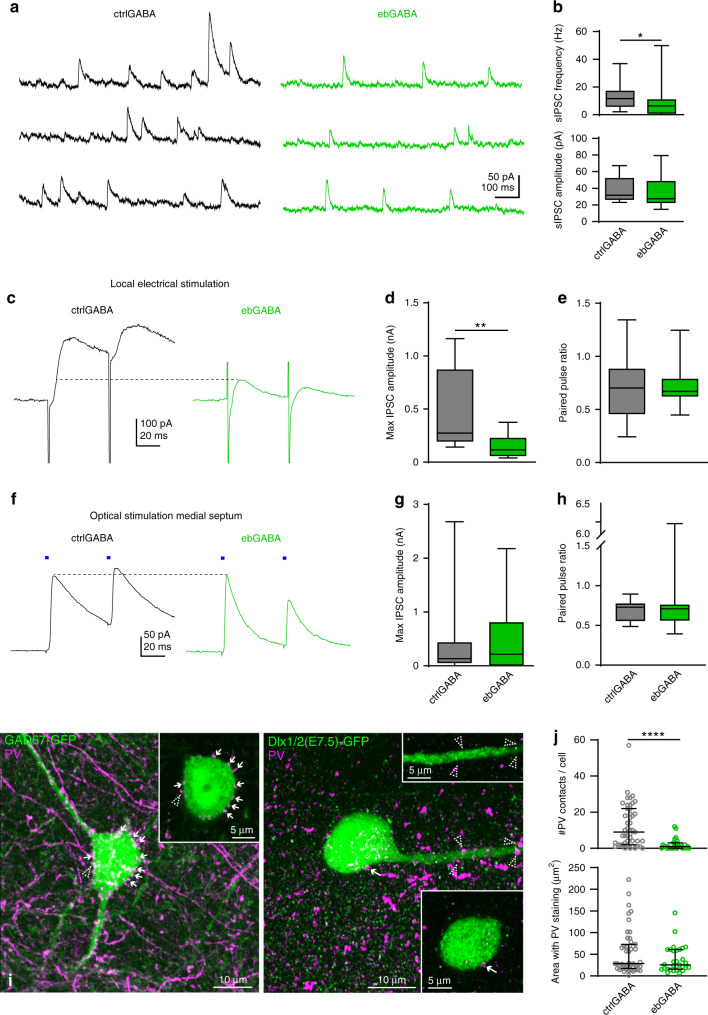
Sparse local inhibitory inputs onto adult ebGABAs. **a** Example voltage clamp traces showing lower sIPSC frequency in a ebGABA cell than in a ctrlGABA cell. **b** sIPSC frequency but not amplitude is significantly lower in ebGABAs (*n* = 17 cells) than ctrlGABAs (*n* = 18 cells; *P* = 0.043, two-sided Mann–Whitney *U* test). **c** Representative IPSCs evoked by local electrical stimulation. IPSCs evoked in the ctrlGABA cell display higher amplitude than the ones evoked in the ebGABA cell. Stimulation artifacts were trimmed for clarity. **d** The maximum amplitude of the IPSC evoked by local electrical stimulation is significantly lower in ebGABAs than ctrlGABAs (*P* = 0.0085, Mann–Whitney *U* test, ctrlGABAs *n* = 10, ebGABAs *n* = 8). **e** IPSC paired pulse ratio is not statistically different between ctrlGABAs and ebGABAs (*P* = 0.792, two-sided Mann–Whitney *U* test, ctrlGABA *n* = 10, ebGABA *n* = 8). **f** Representative IPSCs evoked by optical stimulation of medial septal terminals. IPSCs evoked in ctrlGABA and ebGABA cells display similar amplitudes. **g**, **h** No significant difference between ebGABAs (*n* = 12 cells) and ctrlGABAs (*n* = 13 cells) in maximum amplitude (*P* = 0.611, two-sided Mann–Whitney *U* test) and paired pulse ratio (*P* = 0.796, two-sided Mann–Whitney *U* test) of the IPSCs evoked by optical stimulation of septal terminals. **i** Representative maximum intensity projections of confocal z-stacks showing innervation of a ctrlGABA and an ebGABA by PV+ boutons. Appositions between PV+ boutons and GFP+ somata or dendrites are highlighted with arrows; lack of apposition is marked with dashed arrowheads. Insets: single optical sections (thickness 0.41 µm) showing magnifications of the appositions. Left, z-stack depth 20.5 µm. Right, z-stack depth 15.17 µm. Experiment repeated independently for 47 ctrlGABAs and 30 ebGABAs with similar results. **j** EbGABAs display a significantly lower number of PV+ appositions than ctrlGABAs (top, *P* < 0.0001, two-sided Mann–Whitney *U* test, *n* = 47 ctrlGABAs, *n* = 30 ebGABAs); area of the PV staining is not significantly different between the two populations of cells (bottom, *P* = 0.236, two-sided Mann–Whitney *U* test, *n* = 47 ctrlGABAs, *n* = 30 ebGABAs). Boxplots in panels **b**, **d**, **e**, and **g**, **h** represent medians (center), interquartile ranges (bounds), minima and maxima (whiskers). In panel **j**, data are represented as scatter plots with medians and interquartile ranges. *P < 0.05. **P < 0.01. ****P < 0.0001. Source data are provided as a Source Data file.

Given that ebGABAs are biased toward a deep location and that the radial position influences the inhibitory innervation of CA1 pyramidal cells^24^, we also asked whether an early birth date biases GABA cells toward specific inhibitory wiring schemes. We began by examining sIPSCs by voltage clamping the cells at the reversal potential for glutamatergic currents (0 mV; ctrlGABA *n* = 18, ebGABA *n* = 17, Fig. 5a). As many sIPSCs are action potential-dependent, this measurement largely portrays local inhibition driven by interneurons firing in the slice. We confirmed that sIPSCs were mediated by activation of GABA_A_ receptors as they were completely abolished by the GABA_A_ receptor antagonist Gabazine (SR95531, 10 µM, *n* = 4 cells). EbGABAs displayed a significantly lower sIPSC frequency compared to ctrlGABAs (ctrlGABAs: median 11.7 Hz, IQR: 6.2–16.8 Hz; ebGABAs: median 6.5 Hz, IQR: 1.2–10.6 Hz; *P* = 0.043, Mann–Whitney *U* test). Lower frequency of sIPSCs in ebGABAs could arise from decreased spontaneous activity of interneurons innervating ebGABAs, sparser innervation by GABAergic terminals or lower release probabilities of presynaptic GABAergic terminals. To verify whether weaker innervation by GABAergic terminals underlies this effect, we carried out further experiments.

First, we placed a bipolar stimulating electrode in stratum radiatum, the layer with weakest innervation by medial septal axons (Supplementary Fig. 6b) to bias our stimulation toward interneurons. In line with the sIPSC data, the maximum IPSC evoked by local electrical stimulation was significantly higher in ctrlGABA than ebGABA (*P* = 0.0085, Mann–Whitney *U* test, ctrlGABA *n* = 10, ebGABA *n* = 8, Fig. 5c, d). Furthermore, IPSC paired pulse ratio did not differ between the groups (Fig. 5e). These observations suggest that sparser GABAergic innervation and not weaker release probability at GABAergic synapses could account for the lower inhibitory tone onto ebGABA.

To corroborate that this deficit in inhibition arises from local sources, we probed the GABAergic input from the medial septum, a region that provides significant innervation of CA1 GABAergic cells^25^, by virally driven Chronos expression in this nucleus (Supplementary Fig. 6a). Following at least two weeks of expression, Chronos/tdTomato+ axons densely innervated the stratum oriens and the border between the stratum radiatum and the stratum lacunosum-moleculare (and to a lesser extent the strata pyramidale and radiatum; Supplementary Fig. 6b).

Both ctrlGABA and ebGABA displayed a high degree of connectivity (13/14 ctrlGABA and 12/18 ebGABA receiving IPSCs upon septal stimulation, Supplementary Fig. 6d). These IPSCs were GABAergic as they were blocked by the GABA_A_ receptor antagonist Gabazine (SR95531, 10 µM, 11/11 cells, Supplementary Fig. 6c). Maximum amplitude of the IPSC evoked by septal stimulation as well as paired pulse ratio did not differ between ctrlGABAs (*n* = 13) and ebGABAs (*n* = 12, Fig. 5e, f), suggesting that these cells received similar innervation and release probability from this pathway. Thus, the deficit in inhibition onto ebGABAs is likely to originate from local sources.

### EbGABAs receive sparse innervation from parvalbumin neurons

Parvalbumin-expressing (PV+) basket cells were previously shown to differentially target CA1 pyramidal neurons according to their radial position^24^. Since the radial position of pyramidal cells is highly influenced by their date of birth^26^, we tested whether ebGABAs could also receive different innervation by PV+ cells. To this end, we quantified the number of PV+ boutons innervating ebGABAs and ctrlGABAs. To quantify the innervation ctrlGABAs, we used the GAD67-GFP mouse line. To avoid bias due to uneven sampling across layers, we sampled ctrlGABA cells that roughly matched the position of the imaged ebGABAs (*n* = 43 ctrlGABA and *n* = 30 ebGABA, each population from two mice). We found a striking difference in the number of boutons innervating the two populations, with ebGABAs receiving a significantly lower number of PV+ terminals (*P* < 0.0001, Mann–Whitney *U* test, Fig. 5i, j). Importantly, we verified that intensities and areas of the PV staining, as well as the sampled volumes were similar for the two populations (*P* = 0.2363, Mann–Whitney *U* test, Fig. 5j and Supplementary Fig. 7). In addition, the difference in the number of PV+ terminals held when restricting the analysis to cells in the stratum oriens (*P* = 0.0008, Mann–Whitney *U* test, Supplementary Fig. 7d), suggesting that this difference was not generated by uneven sampling across layers. Thus, an early birth date biases GABA cells for a sparse PV innervation, which is likely to account (at least in part) for the weak inhibition observed in ebGABAs.

### ebGABAs show distinct relation to network activity

**Fig. 6:**
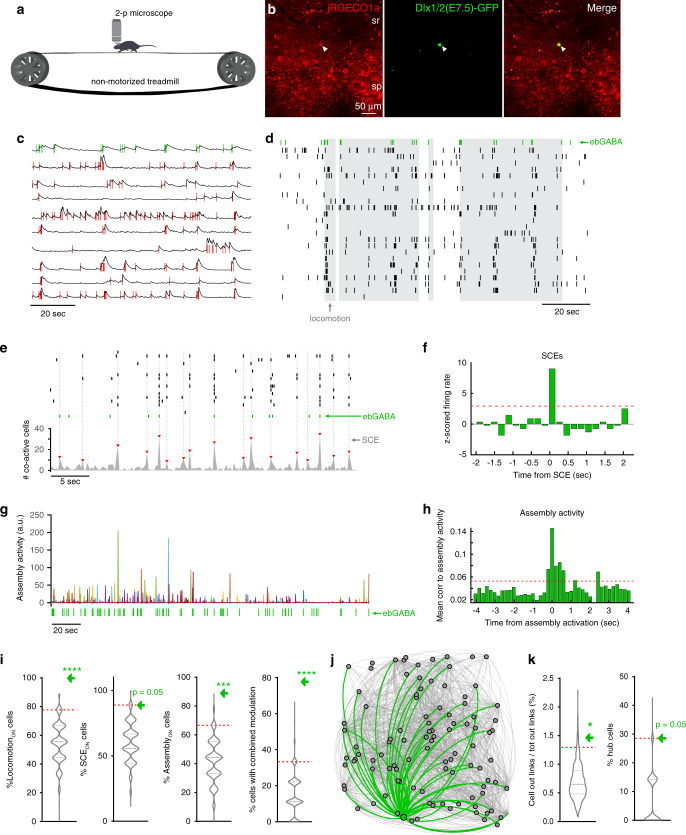
ebGABAs correlate with population synchrony and show high functional connectivity in vivo. **a** Schematic of the experimental configuration. **b** jRGECO1a and Dlx1/2(E7.5)-GFP signals from CA1 stratum pyramidale. Experiment repeated in seven FOVs from six mice with similar results. **c** Normalized Δ*F*/*F* traces and corresponding inferred spikes (vertical lines). Top trace (green spikes): ebGABA cell. Each Δ*F*/*F* trace was filtered and normalized to the maximum value. **d** Raster plot showing the activity of the ebGABA and 23 GFP-negative cells during locomotion (gray areas). **e** Top, raster plot showing the activity of the ebGABA and 19 GFP-negative cells during SCEs (gray vertical lines). Bottom, number of co-active cells in time. Dashed lines denote SCEs. **f** *Z*-scored PSTH showing a significant response to SCEs of the ebGABA. **g** Assembly activities in time during rest. Seven significant assemblies were detected (assemblies are color coded). **h** Mean cross-correlogram between ebGABA spike count and the activity of each assembly showing a correlation above chance level. **i** Proportions of modulated ebGABAs (green arrows, *n* = 9 cells from seven FOVs) in relation to surrogate distributions (matched subsampling, violin plots). EbGABAs showed a higher proportion of modulation by locomotion (*P* < 0.0001), SCEs (*P* = 0.05), assembly activity (*P* < 0.001) and all three phenomena (*P* < 0.0001) than random cells (*n* = 776 cells, two-sided bootstraps). **j** Network graph illustrating functional output connectivity. EbGABA and its output connections are depicted in green. All other cells and their output connections are represented in gray. **k** EbGABAs’ average percentage of functional output connections over the total amount of connections in the FOV (left), and proportion of ebGABAs acting as hub cells (right, green arrows, *n* = 7 cells from five FOVs) in relation to surrogate distributions (matched subsampling, violin plots). EbGABAs showed a higher number of output connections (*P* < 0.05) and a higher proportion of hub cells (*P* = 0.05) than random cells (*n* = 746 cells, two-sided bootstraps). Panels **b**–**h** and **j** refer to the same FOV. Red dashed lines depict statistical thresholds (see Methods for details). Dashed lines in violin plots show quartiles. ***P < 0.001. ****P < 0.0001. sp stratum pyramidale, sr stratum radiatum.

Finally, we asked whether ebGABAs’ different anatomical, electrophysiological and wiring properties reported above could result in distinct in vivo activity in awake mice. To test this, we injected a viral vector expressing the red calcium indicator jRGECO1a in the dorsal hippocampus of adult Dlx1/2(E7.5)-GFP mice. Two weeks after the injections, mice were implanted with a chronic glass window that was placed just above the dorsal hippocampus and a bar for head fixation. This allowed performing in vivo two-photon calcium imaging from ebGABA and nearby cells. Mice were head-fixed in the dark on a non-motorized treadmill allowing spontaneous movement^27^ (Fig. 6a).

We imaged from 15 mice expressing jRGECO1a in large numbers of cells using 400 × 400 µm fields of view (FOVs, Supplementary Movies 2 and 3). Given the sparsity of ebGABAs, only 17 Dlx1/2(E7.5)-GFP+ cells were found out of 15 mice. Analyses of the calcium dynamics during spontaneous locomotion and rest could be performed only for nine ebGABAs and nearby cells (*n* = 776 in total) from seven FOVs from six mice (two FOVs from stratum oriens and six from the stratum pyramidale). The remaining eight ebGABAs could not be analyzed because of either excessive movement in the *z*-axis (four cells), no expression of jRGECO1a in the Dlx1/2(E7.5)-GFP+ cells (three cells) or epileptic-like activity detected in the FOV (one cell). We employed a matched subsampling approach (see “Methods”) to determine statistically whether ebGABAs, as a population, displayed significantly different mean parameters (or proportional modulation) than control cells. The inferred firing rate of ebGABAs was not significantly different from the rate of random control cells (Supplementary Fig. 8a). We next examined single cells’ activity during locomotion (Fig. 6d, see “Methods” for details). We found that all ebGABAs (9/9) were recruited during locomotion (Locomotion_ON_ cells Fig. 6d, i), mostly for the entire locomotion period, but in one case only at locomotion onset (Supplementary Fig. 8b, c). The proportion of Locomotion_ON_ ebGABAs was significantly higher than the proportion of Locomotion_ON_ random control cells (*P* < 0.0001, bootstrap). We also analyzed single cell activities in relation to synchronous calcium events (SCEs, Fig. 6e and Supplementary Fig. 9, see “Methods” for details) that are known to occur during rest, often in synchrony with sharp-wave ripples^28^. SCEs occurred at a rate of 0.08 ± 0.04 Hz in stratum pyramidale (mean ± SD), in line with previous reports, and of 0.02–0.03 Hz in stratum oriens. We found that 8/9 ebGABAs were significantly recruited during SCEs (SCE_ON_ cells, Fig. 6f). This proportion was higher than the proportion of SCE_ON_ random control cells (*P* = 0.05, bootstrap, Fig. 6i).

Next, we detected cell assembly patterns occurring during rest in a 200 ms time window and analyzed single cell activities in relation to assembly activations (Fig. 6g and Supplementary Fig. 9, see “Methods” for details). This analysis revealed that 7/9 ebGABAs were significantly recruited around “mean cell assembly activity” (Assembly_ON_ cells, Fig. 6h). The proportion of Assembly_ON_ ebGABAs was significantly higher than the proportion of random control cells (*P* < 0.001, bootstrap, Fig. 6i).

Overall, 7/9 ebGABAs displayed combined modulation by locomotion, SCEs and assembly activities and this proportion was significantly higher than the proportion of random control cells showing combined modulation (*P* < 0.0001, bootstrap, Fig. 6i). This suggests that ebGABAs constitute an important node of the hippocampal circuit: they are among a minority of cells in the network that integrate both locomotion and population synchrony signals.

Finally, we asked whether ebGABAs exhibit distinct functional connectivity even in adult networks. We analyzed functional connectivity in a subset of FOVs imaged from the stratum pyramidale (five FOVs, seven ebGABA, 746 other cells). We calculated output functional connectivity similarly to our previous studies in developing networks^11,17^. In brief, a functional connection from neuron A to neuron B was established if neuron A consistently fired before B. Although the connectivity of ebGABAs varied across FOVs (percentage of ebGABA connections among all connections: 1.5 ± 1.3%, mean ± SD, Supplementary Fig. 8d), as a population ebGABAs connected to a larger number of cells than random control cells (*P* < 0.05, bootstrap, Fig. 6j, k). In line with this, 2/7 ebGABAs (28%) could be defined as hub cells (see Methods for criteria), whereas the proportion of hub cells among control cells was smaller (60/746, 8%; *P* = 0.05, bootstrap). The difference in functional connectivity between ebGABAs and control cells appeared to be driven by locomotion periods (Supplementary Fig. 8e). Thus, ebGABAs remain crucial nodes of the CA1 network even in adulthood in vivo.

## Discussion

Using inducible genetic fate mapping, ex vivo and in vivo large-scale calcium imaging, electrophysiology, optogenetics, and anatomical analyses, we have shown that ebGABAs are involved in local CA1 dynamics both in development and adulthood. At the neonatal stage, ebGABAs coordinate network bursts (GDPs) ex vivo. In adulthood, ebGABAs maintain a strong link with network activity and high functional connectivity in vivo. Their early birth date specifies anatomical and intrinsic electrophysiological properties as well as input connectivity schemes that may contribute to their recruitment.

We found that ebGABAs maintain a set of distinct anatomical and functional properties in the adult CA1. First, they display sparse local axons and express combinations of markers typical of different classes of projection cells. Electrophysiologically, they are characterized by a small “sag” and a sublinear f/I (input/output) relationship. The latter may limit strong activations to short periods of time. In addition, adult ebGABAs show wiring schemes that are distinct from randomly sampled GABAergic cells. Their recruitment appears to be largely driven by intra-hippocampal excitatory afferents because they receive typical intra-hippocampal excitation, but little long-range inputs from the EC and the VMT. Furthermore, ebGABAs receive weak local inhibition via a sparse innervation by axons arising from PV+ neurons. In vivo, ebGABAs are part of a minority of cells in the CA1 network that are recruited with locomotion, synchronous calcium events and assembly activity. In addition, they exhibit high functional output connectivity degrees. Therefore, ebGABAs appear predetermined for exceptional functional and structural properties in both the developing and adult hippocampus.

The present study demonstrates that ebGABAs are highly involved in hippocampal network dynamics in development and adulthood. During early postnatal development, stimulation of a single ebGABA is sufficient to trigger GDPs and to change their frequency. The latter phenomenon is likely to involve complex polysynaptic interactions because the delay between ebGABA stimulation and GDP onset ranged between 0.2 and 5 s. Thus, hub cells are not a unique feature of CA3, which shows more recurrent connections^29^, but are distributed throughout the hippocampal formation, and possibly throughout the brain. It is not clear whether CA1 hub cells orchestrate GDPs only through action on the CA1 circuit or, by contrast, they modulate GDP generation in CA3 with subsequent propagation to CA1. Both scenarios are possible. Reconstructions of biocytin-filled ebGABAs demonstrated that some projected back to CA3, a finding that is corroborated by our analyses of molecular marker combinations (i.e., strong nNOS-expressing backprojection cells). On the other hand, GDPs have been shown to occur in CA1 also independently from CA3^13,30^.

GDPs and their in vivo counterpart^31^ are network bursts that could represent an early form of sharp wave ripple (SWRs). SWRs are generated in CA2 and, similarly to GDPs, require the recurrent circuit of CA3 to successfully propagate to CA1^32,33^. Recently, we reported that SCEs detected with calcium imaging in the adult CA1 often occur during SWRs and involve reactivations of cell assemblies^28^. Here, we show that ebGABAs maintain a strong relationship with network bursts (SCEs) in adulthood.

In addition, these cells are consistently recruited around the activation of CA1 assemblies. This may be achieved via special intrinsic properties and circuit motifs. For instance, we have shown with ex vivo patch clamp experiments that ebGABAs display efficient rate coding for short but not long depolarizing stimuli, a mechanism that could favor their transient recruitment in vivo. In addition, our data suggest that ebGABAs receive the majority of their excitatory inputs from intra-hippocampal inputs. This may enhance their signal to noise ratio to report local network activity to postsynaptic targets. If such intra-hippocampal inputs are most likely originating from CA3 (since they are evoked by electrical stimulation in the *stratum radiatum*), future experiments should also probe the contribution of synaptic inputs from CA1 and CA2 pyramidal cells to the excitation of ebGABAs. A preferential input from CA2 could be expected, given that neurons sharing a similar temporal embryonic origin are more likely to connect^34,35^ and that CA2 is the earliest region of the *Cornus Ammonis* to be generated^36^. Preferential CA2 inputs could also provide a circuit mechanism for the high modulation of ebGABAs by assembly activation, given the role of CA2 in triggering SWRs^33^.

Finally, we demonstrate that ebGABAs receive little local inhibition from PV+ interneurons, but high levels of long-range inhibition from the medial septum. This finding is consistent with a study showing that the majority of inhibitory terminals on CA1 GABAergic projection cells arise from the medial septum^37^. Virtually all PV+ terminals originating from CA1 interneurons and targeting other GABA cells are likely to arise from PV+ basket cells and bistratified cells, and both cell types are strongly active during SWRs^38,39^. Thus, this lack of cell type-specific inhibition could additionally favor ebGABAs’ recruitment during SWRs/SCEs. Since superficial pyramidal cells receive little PV innervation but abundant inputs from CCK+ interneurons^35^, future work could establish whether ebGABAs receive preferential innervation from CCK+ interneurons.

Another remarkable feature of ebGABAs in the adult CA1 in vivo is their functional versatility, namely their recruitment during a variety of behavioral/network phenomena. Specifically, ebGABAs were among a minority of cells in the network that were consistently activated with locomotion, SCEs and assembly activations. This versatility sets them apart from most known GABAergic cell types because interneurons that are activated by locomotion are usually not activated during SWRs, and vice versa^38–40^.

Interestingly, ebGABAs’ recruitment during a variety of network states is reminiscent of the activity described for CA1 GABAergic projection neurons^41^. In line with this, a significant portion of ebGABAs project to the medial septum^12^. In the present study, we have found that ebGABAs express combinations of molecular markers typical of two extra classes of GABAergic projection cells: strong nNOS-expressing backprojection cells, likely innervating dentate gyrus and CA3^14,19^, and M2R-expressing retrohippocampal cells that innervate subiculum and retrosplenial cortex^20^. Combined with the fact that biocytin-filled ebGABAs exhibited little local axonal arborization, these data indicate that ebGABAs could form several classes of projection cells innervating various target regions. EbGABAs also share some similarities with the recently described long-range inhibitory nNOS+ cells (LINCs)^42^, in particular laminar location and various long-range targets. However, overlap between these two classes could be small because LINCs are born later (E11.5) and rarely express SOM.

EbGABAs that we filled with biocytin in coronal slices from adult mice showed poor local axonal arborizations. However, ebGABAs displayed strong functional output connectivity in the adult CA1 in calcium imaging experiments in vivo. Various reasons could lie at the bottom of this apparent discrepancy. EbGABA’s axons could innervate CA1 cells through arborizations in different planes that are largely severed in acute coronal slices. Alternatively, local projections could be minimal but circuit *motifs* could guarantee a powerful effect on the CA1 network. These could be local disinhibition (i.e., inhibition of few CA1 interneurons targeting many pyramidal cells, such as basket cells) or inhibition of distant regions that project to CA1 (such as the medial septum or CA3).

We report that an early embryonic origin results in a deep soma location, sublinear input-output firing curve, small sag, reduced innervation by PV+ interneurons and by long-range excitatory inputs, in particular by thalamic afferents. These findings highlight that birth date and/or radial position may have a similar impact on certain cellular features for GABAergic and glutamatergic neurons. We recently reported that a low input–output firing curve also distinguishes dentate gyrus granule cells with an early temporal embryonic origin from later-born granule cells^43^. In addition, deep CA1 pyramidal cells are born earlier than superficial pyramidal cells^26^ and show a smaller sag^24,44^. However, deep CA1 pyramidal cells show the opposite pattern of PV innervation from ebGABAs, receiving more PV+ inputs than superficial cells^24^.

An early embryonic origin specifies a large proportion of long-range projecting GABAergic cells, an extremely rare neuronal population^22^. It is crucial to understand how this early specification occurs. Early transcription factors shared across different subpallial proliferative areas could direct GABAergic cells toward a long-range projecting fate. It was recently proposed that COUP-TFII acts as a temporal identity cue that promotes an early specification toward SOM+ GABA neurons^21^, however, very few Dlx1/2(E7.5)-GFP+ cell expressed it in adulthood.

Our study has some limitations. First, the number of recorded ebGABAs in some experiments is low. This is due to the sparseness of GFP+ cells in the hippocampus of our Dlx1/2(E7.5)-GFP mouse line. This sparseness reduces the yield of most experiments. The low number of ebGABAs is consistent with the scantiness of GABAergic projection cells^22^. In addition, our Dlx1/2(E7.5)-GFP line is unlikely to capture all hippocampal ebGABAs. Importantly, we have kept tamoxifen levels low and used a weak reporter line because this minimizes known “leak” issues of the Dlx1/2-Cre^ER^ line^12^. Second, future studies should investigate ebGABAs’ axonal fields filled in vivo and their postsynaptic targets. Furthermore, they should test whether these cells are causally involved in adult network activity. Intersectional genetic strategies should be developed to tackle these questions because Cre is no longer expressed in adult ebGABAs, leading to inability to target these neurons with Cre-dependent constructs.

On balance, this study shows that ebGABAs are pioneer GABAergic cells operating as “hubs” during development and maintaining unique connectivity throughout adulthood. To our knowledge, we have provided the first evidence that an early birth date alone (regardless of spatial embryonic origins or cell types) dictates anatomical, electrophysiological and connectivity properties of GABAergic cells. Given their bias toward long-range targets, intra-hippocampal inputs and local assembly activity, we hypothesize that ebGABAs could detect CA1 activity and bind local and distant assemblies into chains of neuronal activation^45^.

## Methods

### Animals

All protocols were performed under the guidelines of the French National Ethics Committee for Sciences and Health report on “Ethical Principles for Animal Experimentation” in agreement with the European Community Directive 86/609/EEC under agreement #01413. Dlx1/2Cre^ER+/−^;RCE:LoxP^+/+^ male mice^15^ were crossed with 7- to 8-week-old wild-type Swiss females (C.E. Janvier, France) for offspring production. To induce Cre^ER^ activity, we administered a tamoxifen solution (Sigma) by gavaging (force-feeding) pregnant mice with a silicon-protected needle (Fine Science Tools). We used 2 mg of tamoxifen solution per 30 g of body weight prepared at 10 mg/mL in corn oil (Sigma). In order to label neuronal progenitors expressing Dlx1 and/or Dlx2 with GFP at embryonic age E7.5 or E8.5, pregnant females crossed with Dlx1/2Cre^ER+/−^;RCE:LoxP^+/+^ males were force-fed at day 7.5 or 8.5 post vaginal plug. For simplicity, we refer to Dlx1/2Cre^ER+/−^;RCE:LoxP^+/+^ mice treated with tamoxifen at E7.5/E8.5 as Dlx1/2(E7.5)-GFP. GAD67-GFP mice were kindly donated by Dr. Hannah Monyer (Heidelberg University).

### Slice preparation for ex vivo experiments

Slices containing the hippocampus were prepared from Dlx1/2(E7.5)-GFP mice or Cre-negative littermates Dlx1/2Cre^ER−/−^;RCE:LoxP^+/+^. Four to 5 days old (P4–P5) pups were used for developmental experiments and 4–33 weeks old mice (mean age ± SD: P67 ± 32) for adult experiments. Adult mice were anaesthetized with a ketamine/xylazine mix (Imalgene 100 mg/kg, Rompun 10 mg/kg) or a Domitor/Zoletil mix (0.6 and 40 mg/kg, respectively) prior to decapitation. Slices were cut using a Leica VT1200 S vibratome in ice-cold oxygenated modified artificial cerebrospinal fluid with the following composition (in mM): 2.5 KCl, 1.25 NaH_2_PO_4_, 7 MgCl_2_, 5 CaCl_2_, 26 NaHCO_3_, 5 d-glucose, 126 CholineCl. Slices were cut in the horizontal plane at 380 µm thickness for developmental experiments and in the coronal plane at 300 µm thickness for adult experiments. Slices were then incubated in oxygenated normal ACSF containing (in mM): 126 NaCl, 3.5 KCl, 1.2 NaH_2_PO_4_, 26 NaHCO_3_, 1.3 MgCl_2_, 2.0 CaCl_2_, and 10 d-glucose (1 h at room temperature, RT for developmental experiments, 15 min at 33 °C followed by 30 min at RT for adult experiments).

### Ex vivo calcium imaging and patch clamp recordings during development

For Fura2-AM loading, slices were incubated in a small vial containing 2.5 ml of oxygenated ACSF with 25 ml of a 1 mM Fura2-AM solution (in 100% DMSO) for 30 min. Slices were incubated in the dark, and the incubation solution was maintained at 30–33 °C. Slices were transferred to a submerged recording chamber and continuously perfused with oxygenated ACSF (3 mL/min) at 30–33 °C. Imaging was performed with a multibeam multiphoton pulsed laser scanning system (LaVision Biotech) coupled to a microscope as previously described^46^. Images were acquired through a CCD camera, which typically resulted in a time resolution of 80–138 ms per frame. Slices were imaged using a 20×, NA 0.95 objective (Olympus). Imaging depth was on average 80 µm below the surface (range: 50–100 µm).

Patch recording electrodes (4–8 MΩ resistance) were pulled using a PC-10 puller (Narishige) from borosilicate glass capillaries (GC150F-10, Harvard Apparatus) and filled with a filtered solution consisting of the following compounds (in mM): 130 K-methylSO4, 5 KCl, 5 NaCl, 10 HEPES, 2.5 Mg-ATP, 0.3 GTP, and 0.5% neurobiotin (265–275 mOsm, pH 7.3). Electrophysiological signals were amplified (EPC10 amplifier; HEKA Electronik), low-pass filtered at 2.9 kHz, digitized at 10 kHz and acquired using a Digidata 1550 Digitizer and pClamp 10 software (Molecular Devices). For most stimulation experiments, imaging acquisition was separated between: (1) a baseline period during which the cell was held close to resting membrane potential (i.e., zero current injection); (2) a stimulation period during which phasic stimulation protocols were applied; and (3) a 3 min recovery period during which the cell was brought back to resting membrane potential. The stimulation protocol consisted of suprathreshold current pulses (amplitude: 100–150 pA) of 200 ms duration repeated at 0.1, 0.2, and 0.4 Hz. Cells were discarded if they did not meet the following criteria: (1) stable resting membrane potential; (2) stable network dynamics measured with calcium imaging (i.e., the coefficient of variation of the inter-GDP interval did not exceed 1); (3) cells displaying healthy shape and good Fura2-AM loading throughout the entire field of view.

### Analysis of ex vivo calcium imaging data during development

We used custom designed MATLAB software allowing automatic identification of loaded cells, measurement of the average fluorescence transients from each cell as a function of time and detection of onsets and offsets of calcium signals^11^. Network synchronizations (GDPs) were detected as synchronous onsets peaks including more neurons than expected by chance, as previously described^11^, and their time stamp denoted by *t*_G_. The “Inter GDP intervals” (IGI) was defined as the interval between two consecutive GDPs. To establish whether the stimulation of a single neuron was able to influence the frequency of GDPs occurrence, we employed a previously published analysis^11^. We first calculated the average IGI in the three epochs: pre-stimulus (control), during the stimulation period and post-stimulus. Due to the variability distribution of IGI in each interval, we calculated the average IGI in a window of *t*_*s*_ (300 or 400 frames calculated starting from each t_*G*_ and eliminating the data corresponding to overlaps between epochs). To test for the significance of the change in the period of GDP, a Kolmogorov–Smirnov test was applied between all the three resulting distributions of average IGI and a significance level of *P* < 0.05 was chosen. To test for changes in phase of the GDP upon stimulation, the IGI during resting conditions was used as a reference period of a harmonic oscillator mimicking the rhythm of GDPs. The phase of the expected GDP generated by the harmonic oscillator was compared to the phase of the real GDP intervals occurring during the entire recording. For the *i*th GDP, a phase measure *Φ*_*i*_ in respect to the control IGI is defined as follows:

1 $${\mathrm{{\Phi}}}_i = \frac{{(t_G^i - < t_G^i > )}}{{{\Delta}t}},$$

where Δ*t* is the average IGI interval in the control condition, and 〈$t_G^i$〉 = *i* × Δ*t* is the expected occurrence of the *i*th GDP, according to the control condition. The phase of each real GDP was set to zero at the peak of synchronous activation and increased linearly reaching 2*π* at the peak of the next GDP. By subtracting the phase of real GDPs from the expected GDP, we could instantaneously measure the effect of phasic stimulation. The cell was included in the dataset only if the difference between expected and observed cycles did not exceed ±2 cycles during resting conditions.

To assess whether single cell stimulation was able to lock GDP occurrence, we isolated the trials following each stimulation (trial length was either 2.5 or 5 s). Next, we calculated an average histogram of single cell calcium onsets including all trials. The histogram was then normalized by the number of GDPs during the stimulation protocol. The height of the highest peak in the histogram was stored. To estimate the significance of the time locking, we created surrogate data consisting of concatenated control and post-stimulation frames (without stimulation trials). The same histogram was then produced from the surrogate data. Since no stimulation was present in this case, we used an arbitrary frame as stimulation time. For each cell, number of trials and trial length for the surrogate data matched the ones of stimulation data. This process was repeated 100 times using 100 different starting frames for the stimulation, and the values of the highest peaks were stored. A final histogram of peak heights was computed, and the cell was considered to significantly lock GDP onset if the peak height during stimulation fell within the 5% highest peaks in the distribution (i.e., *P* < 0.05).

### Virally-driven opsin/jRGECO1a expression

Dlx1/2(E7.5)-GFP mice (P25–P35 age for opsin expression, 2–3 months age for jRGECO1A expression) were anaesthetized using either a ketamine/xylazine mix (Imalgene 100 mg/kg, Rompun 10 mg/kg) or 1–3% isoflurane in oxygen. Analgesia was also provided with buprenorphine (Buprecare, 0.1 mg/kg). Lidocaine cream was applied before the incision for additional analgesia. Mice were fixed to a stereotaxic frame with a digital display console (Kopf, Model 940). Under aseptic conditions, an incision was made in the scalp, the skull was exposed, and a small craniotomy was drilled over the target brain region. A recombinant viral vector was delivered using a glass pipette pulled from borosilicate glass (3.5” 3-000-203-G/X, Drummond Scientific) and connected to a Nanoject III system (Drummond Scientific). The tip of the pipette was broken to achieve an opening with an internal diameter of 25–35 μm. The following viral vectors were used (both from Penn Vector Core): AAV8.Syn.Chronos-tdTomato.WPRE.bGH to drive Chronos expression in the VMT or medial septum, AAV1.Syn.NES.jRGECO1a.WPRE.SV40 to drive jRGECO1a expression in the dorsal CA1 (for the latter, virus stock was diluted 1:4 in d-phosphate-buffered saline (PBS), Sigma Aldrich). Stereotaxic coordinates were based on a mouse brain atlas (Paxinos and Franklin). All coordinates are in millimeters. Anteroposterior (AP) coordinates are relative to bregma; mediolateral (ML) coordinates are relative to the sagittal suture; dorsoventral (DV) coordinates are measured from the brain surface. For EC, VMT (nuclei reuniens and rhomboid), and medial septum, 100 nL of virus were injected at a rate of 20 nL/min at the following coordinates: EC −4.7 AP, −4.1 ML, −2.7 DV; VMT −0.7 AP, 0 ML, −3.8 DV; medial septum +0.9 AP, 0 ML, −3.8 DV. For CA1 hippocampus, two injections of 250 nL were performed at a rate of 25 nL/ min (−1.8 AP, −1.6 ML, −1.25 DV and −2.3 AP, −2.4 ML, −1.25 DV). EC injections mostly targeted the lateral EC. At least 2 weeks were allowed for Chronos and jRGECO1a expression before recording procedures and chronic hippocampal window, respectively.

### Ex vivo patch clamp recordings and optogenetics in adulthood

Patch clamp recordings in adult slices were performed using a SliceScope Pro 1000 rig (Scientifica) equipped with a CCD camera (Hamamatsu Orca-05G). Slices were transferred to a submerged recording chamber and continuously perfused with oxygenated ACSF (3 mL/min) at ~32 °C. Patch recording electrodes (4–6 MΩ resistance) were produced as described above. For current clamp recordings, electrodes were filled with an intracellular solution containing (in mM): 125 k-gluconate, 5 KCl, 4 ATP-Mg, 0.3 GTP-Na_2_, 10 Na_2_-phosphocreatine, 10 HEPES and 0.05% neurobiotin (pH 7.3 and ~280 mOsm). For voltage clamp experiments, an intracellular solution with the following ingredients (in mM) was used: 152 Cs-methanesulfonate, 4 CsCl, 4 ATP-Mg, 0.3 GTP-Na_2_, 10 Na_2_-phosphocreatine, 10 HEPES (pH 7.3 and ~280 mOsm). Electrophysiological signals were amplified (Multiclamp 700B), low-pass filtered at 2.9 kHz, digitized at 5–20 kHz and acquired using a Digidata 1440 A digitizer and pClamp 10 software (all from Molecular Devices).

Patch clamp recordings were performed from visually identified neurons (GFP+ cells for ebGABAs, random putative GABAergic cells for ctrlGABAs). EbGABAs and ctrlGABAs were sampled in roughly equal numbers from all layers of both the dorsal and the intermediate/ventral CA1 to avoid biases toward specific cell types or inputs. We used the following criteria to ensure that no pyramidal cell was included in the ctrlGABA group: first, we avoided pyramidal-shaped neurons in the strata pyramidale and oriens; second, we verified in current clamp experiments that all targeted cells (21/21) displayed stereotypical GABA cell firing; third, we verified histologically a subset of ctrlGABAs that were filled with neurobiotin (*n* = 28, see below for details), and all of them exhibited GABA cell anatomical features.

An optoLED system (Cairn Research) consisting of two LEDs was used to visualize fluorescence signals and stimulate Chronos-expressing afferents in CA1. A 470 nm LED coupled to a GFP filter cube was used to visualize GFP-expressing cells and activate Chronos-expressing terminals (3 ms-long light pulses). A white LED coupled to a TRITC/Cy3 filter cube was used to visualize the Chronos/tdTomato-expressing axons. Cells were tested for postsynaptic responses and included in connectivity data only if located in a part of CA1 densely innervated by tdTomato+ axons. Light was delivered using a 40× objective, leading to a light spot size of ~1 mm, which was able to illuminate all CA1 layers.

Electrical stimulation of the Schaffer collateral and evoked local IPSC experiment were performed by placing a bipolar stimulating electrode made by two twisted nichrome wires in the stratum radiatum of CA1 (between the recorded cell and CA3). The bipolar electrode was connected to a DS2A Isolated Voltage Stimulator (Digitimer Ltd.) delivering 0.2 ms-long stimuli. Postsynaptic current amplitude and paired pulse ratio were assessed by two stimulations separated by 50 ms. Sweeps were separated by 20 s delay to avoid the induction of plasticity and ensure stable responses. Maximum PSCs were determined by delivering increasing stimulation powers and constructing stimulation power vs. PSC amplitude curves. Since these curves usually saturated, the maximum PSC amplitude was measured from the first PSC of the plateau. In the few cases in which the amplitude did not saturate, the response obtained from the maximum stimulation power was used to calculate the maximum amplitude.

For sPSCs experiments, *R*_*s*_ was not compensated because this allowed to monitor more precisely its changes throughout the experiment. For evoked PSC experiments, 60% compensation was applied to the *R*_*s*_. Only recordings with *R*_*s*_ < 30 MΩ were included in the dataset. The cell was discarded if *R*_*s*_ changed by more than 20% throughout the protocol. For electrically evoked and optically evoked PSCs, PSC onset delays were consistent with monosynaptic responses (3.3 ± 1.6 ms, mean ± SD).

### Analysis of ex vivo patch clamp recordings in adulthood

Analysis of intrinsic membrane properties was performed using custom-made MATLAB scripts. The resting membrane potential was estimated by averaging a 60 s current–clamp trace recorded at 0 pA holding current. The input resistance was calculated from the slope of steady-state voltage responses to a series of subthreshold current injections lasting 500 ms (from −50 pA to last sweep with a subthreshold response, 5 or 10 pA step size). The membrane time constant (*τ*) was estimated from a bi-exponential fit of the voltage response to a −30 pA hyperpolarizing pulse. The membrane capacitance was calculated as the ratio between membrane *τ* and input resistance. The first spike in response to a juxta-threshold positive current injection was used to determine: the threshold potential (the first point > 0.5 in the first derivative), the fast afterhyperpolarization (calculated from the threshold potential), the action potential half-width (the width at half-amplitude between the threshold potential and the peak of the action potential). The rheobase (in pA) was determined as a 50 ms current injection, able to generate a spike in 50% of the cases in 10 trials. A 1 s-long current injection of 2× the rheobase was used to determine: the firing rate, the adaptation index (range: 0–1, defined as the ratio between the mean of first three and the last three inter-spike intervals and the burst index (the ratio between the number of spikes in the first 50 ms and the entire depolarizing step). The sag ratio was calculated by injecting a 1 s-long negative current able to hyperpolarize the cell between −100 and −120 mV, and by dividing the amplitude of the hyperpolarization at the end of the current step by the peak of the hyperpolarization at the beginning of the step (sag ratio close to 0 implies high sag, whereas sag ratio close to 1 implies little or no sag). The maximum firing rate was defined as the maximum value reached in a f/I curve with positive current injections from +20 to +820 pA (step size: 50 pA).

Analysis of spontaneous postsynaptic currents (sPSCs) was performed with MiniAnalysis (Synaptosoft). PSC parameters were calculated from 2 min recording time. The decay time (10–90%) was calculated from a mono-exponential fit of mean sPSCs. Analysis of evoked postsynaptic current data were performed with Clampfit 10 (Molecular Devices) and MATLAB custom-made scripts. Since membrane and synaptic parameters may be affected by age, for all adult datasets we tested that the distributions of the ages of the mice used did not differ statistically between ctrlGABA and ebGABA. Additionally, for optogenetics experiments we verified that the time of opsin expression did not differ between the groups.

### In vivo calcium imaging in adulthood

A chronic cranial window was implanted using previously published procedures^27^. Mice were head-fixed on a non-motorized treadmill allowing self-paced locomotion (adapted from ref. ^47^) All experiments were performed in the dark. No reward was given. After three to five habituation sessions, mice were alert but calm and alternated between periods of locomotion and rest during imaging. The treadmill was made of a 180 cm black velvet seamless belt lacking tactile or visual cues mounted on two wheels. The movement of the treadmill was monitored using two pairs of LEDs and photo-sensors that read patterns from a disk attached to one of the wheels, similarly to what previously described^27,48^.

For all experiments, extra sound, odor, touch, and light were minimized during the imaging session. Imaging was performed with a single beam multiphoton laser scanning system coupled to a microscope (TriM Scope II, Lavision Biotech). The Ti: sapphire excitation laser (Chameleon Ultra II, Coherent) was operated at 1030 nm for jRGECO1a excitation and 920 nm for GFP excitation. Fluorescence emission was acquired using a 16× objective (Nikon, NA 0.8) and split in two detectors (GaSP PMT, H7422-40, Hamamatsu) with bandpass filters of 510/10 nm for GFP and 580/20 nm for jRGECO1a. Scanner and PMTs were controlled by a commercial software (Imspector, Lavision Biotech). To optimize the signal-to-noise ratio of fluorescence variation, we used a dwell time exposition of 1.85 µs and a spatial resolution of 2 µm/pixel that allowed us to acquire at 9.85 Hz at a field of view of 400 × 400 µm. Locomotion and imaging triggers were synchronously acquired and digitized using a 1440A Digidata (Axon instrument, 2 kHz sampling) and the pClamp 10 software (Molecular Devices).

### Analysis of in vivo calcium imaging data in adulthood

In vivo calcium movies were pre-processed using the CaImAn toolbox for MATLAB^49^. First, movies were motion-corrected using a rigid registration method^50^. Then, contours and calcium transients were detected using a constrained nonnegative matrix factorization framework allowing denoising and demixing of fluorescence signals^51^. To ensure correct segmentation of somatic calcium activity, the automatic detection was manually refined by adding and removing region of interests (ROIs) using the correlation image based on neighboring pixels. Δ*F*/*F* fluorescence signals were then denoised with a median filter, detrended and extraction of the 20% Δ*F*/*F*. An additional manual refinement was carried out with visual inspection of each ROI in the correlation image and the corresponding trace. Unstable or noisy traces were removed because this led to spurious spike inference. A Markov Chain Monte Carlo approach^52^ initialized by the fast OOPSI algorithm^53^ was used to model rise and decay time constants using a second order autoregressive process, which allowed spike inference from the fluorescence traces. A final visual inspection of overlapping calcium traces and spike raster plots was performed to ensure that the spike times reflected the dynamics of the fluorescence traces.

Temporal alignment of the treadmill movement signal and spike raster as well as subsequent analyses were performed using custom-made MATLAB scripts. Locomotion epochs were defined as time periods with deflections in the photo-sensors signal reading the treadmill movement. Rest epochs were defined as periods >200 ms without treadmill movement. Two methods were used to define cells activated by locomotion. In the first method, peri-stimulus time histograms for the locomotion onset (PSTH^LOC^) were generated (10 s window). The mean $\left( {\mu _{\mathrm{B}}^{{\mathrm{LOC}}}} \right)$ and the SD $\left( {{\upsigma }}_{\mathrm{B}}^{{\mathrm{LOC}}} \right)$ of the baseline firing rate (activity preceding 200 ms before locomotion onset) were used to generate *Z*-score normalized PSTHs:

2 $${Z}^{{\mathrm{LOC}}} = \frac{{{\mathrm{PSTH}}^{{\mathrm{LOC}}} - \mu _{\mathrm{B}}^{{\mathrm{LOC}}}}}{{{\upsigma }}_{\mathrm{B}}^{{\mathrm{LOC}}}}.$$

Cells were defined as significantly activated if at least two consecutive bins exceeded a *Z*-score of 2 in a time window from 200 ms before locomotion onset onwards. This method defined cells activated around the onset of locomotion. In the second method, spike times were circularly shifted to disrupt their temporal relationship with locomotion and rest periods. The ratio between the number of spikes occurring during locomotion and during rest was calculated for each cell of the original and the reshuffled spike matrices. The 95th percentile of the vector containing these ratios for the reshuffled spike times was used as statistical threshold for each cell. Cells were defined as activated by locomotion if their locomotion/rest spike ratio exceeded this threshold. This method defined cells that were more active during locomotion than rest (but not necessarily activated at the onset of locomotion). For each imaging session, a cell was defined as activated by locomotion if it passed at least one of the above tests.

Synchronous calcium events were detected from rest periods binning the spike raster matrix with a 200 ms window. We circularly shifted each spike vector, hence maintaining each cell’s firing rate, but disrupting its temporal relationship to the rest of the population. One thousand surrogate distributions were created. The spikes of each frame for these distributions were computed and the 99th percentile of the resulting “sum of spikes” vector was used as a statistical threshold. Peaks above the threshold that were at least separated by one second were considered SCEs. To define cells activated during SCEs, PSTHs for SCE onset (PSTH^SCE^) were constructed (four seconds window). The mean $\left( {\mu _{\mathrm{B}}^{{\mathrm{SCE}}}} \right)$ and the SD $\left( {{\upsigma }}_{\mathrm{B}}^{{\mathrm{SCE}}} \right)$ of the baseline firing rate (activity preceding SCEs) were used to generate *Z*-score normalized PSTHs

3 $${Z}^{{\mathrm{SCE}}} = \frac{{{\mathrm{PSTH}}^{{\mathrm{SCE}}} - \mu _{\mathrm{B}}^{{\mathrm{SCE}}}}}{{{\upsigma }}_{\mathrm{B}}^{{\mathrm{SCE}}}}.$$

Cells were defined as significantly activated if the bin at zero time lag exceeded a *Z*-score of 3.

Neuronal assemblies (co-active neurons) were detected using an unsupervised statistical method based on independent component analysis^54^. We chose this method because it allows the analysis of assembly activities over time. Analysis was restricted to rest periods and a 200 ms time binning was used because assembly activations coincident to sharp-wave ripples are known to occur in these conditions^28^. Binned spike counts were convolved with a Gaussian kernel and then *Z*-scored to reduce the influence of firing rates. The number of significant co-activation patterns was estimated as the number of principal component variances above a threshold derived from the circularly shifted spike count matrix. Assembly patterns were then extracted with an independent component analysis. To track the activation of cell assemblies over time, a projection matrix was constructed for each pattern from the outer product of its weight vector.

The activity of each assembly was estimated by projecting the columns of the spike matrix (time bins) onto the axis spanned by the corresponding assembly pattern (principal components). The length of the projection was calculated by taking the inner product between the assembly pattern and a weighted sum of the *Z*-scored spike counts^54^.

To define cells significantly modulated by assembly activities, we cross-correlated the spike count vector of each cell to the activity vector of each assembly (8 s time window). For each cell, we averaged cross-correlograms for different assemblies to define ‘mean cross-correlations to assembly activity’. To calculate a statistical threshold for mean cross-correlations, the same procedure was performed on reshuffled data (circularly shifted spike count matrix) and the 95th percentile of this surrogate distribution was set as threshold for each cell. Mean cross-correlograms with at least two consecutive bins out of the five bins around zero time lag exceeding this threshold were defined as significant.

Functional connectivity analysis was performed in Python using our CICADA toolbox (https://gitlab.com/cossartlab/cicada/). This analysis was restricted to FOVs imaged from the stratum pyramidale (*n* = 5, including 7 ebGABAs and 776 control cells) because these had more cells (77–236) than movies recorded from the stratum oriens (*n* = 2, 8–24 cells). The principle of output functional connectivity is to establish a connection from cell A to cell B if the firing of cell A precedes in a repetitive way the firing of cell B. Given the spike times of neuron A, we calculated the distribution of spikes of neuron B occurring at different time lags (between −1000 and +1000 ms). This measure is equivalent to the PSTH of cell B but centered on the firing onsets of cell A. Zero delay correlations were discarded by excluding normal distributions (Gaussians centered at zero) identified using the D’Agostino and Pearson’s test with a two-sided chi squared probability threshold of 0.05 (normaltest function in Python). We also excluded cases in which the activation of two neurons was completely uncorrelated (uniform distributions, identified using the Kolmogorov–Smirnov test from the Python “scipy” package with a two-tailed *P* value threshold of 0.05). A connection was drawn from A to B if the average time lag was greater than zero, or from B to A if it was lower than zero. This procedure was applied to all possible pairs of neurons. The network graph was built with the “Networkx” package in Python. Hub cells were defined based on the following criteria: (1) being functionally connected to at least 5% of the cells in the network; (2) being among the 5% most connected cells in the distribution including cells from all movies; (3) being among the cells with betweenness centrality above the 80th percentile in the distribution including cells from all movies.

To test whether ebGABAs displayed significantly higher proportions of modulation than control neurons, we employed a matched subsampling (bootstrapping) approach. We sampled randomly picked GFP-negative cells to match the amount of ebGABAs in each FOV. This procedure was repeated 1000 times to create a surrogate distribution. The proportion of modulated ebGABAs was considered significantly higher than the proportion of control cells if the former fell above then 95th percentile of the surrogate distribution. The same procedure was employed to test for significance for the output functional connections and the proportion of hub cells.

### Histological processing

For analysis of neurochemical markers expressed in ebGABAs, mice were deeply anaesthetized with a mix of Domitor and Zoletil (0.6 and 40 mg/kg, respectively), then transcardially perfused with 0.1 M PBS followed by 4% PFA in 0.1 M PBS. Brains were post-fixed overnight at 4 °C in 4% PFA in 0.1 M PBS. Brains were then sectioned using a vibratome (VT 1200 s, Leica) into 70–80 μm-thick slices. Sections were stored in 0.1 M PBS containing 0.05% sodium azide until further usage.

Slices containing neurobiotin-filled cells were fixed overnight at 4 °C in 4% PFA in 0.1 M PBS, rinsed in PBS containing 0.3% Triton X-100 (PBST) and incubated overnight at room temperature in streptavidin −488, −555, −594, or −649 (1:1000 in PBST). Imaging was performed using a confocal microscope (Leica TCS SP5-X) equipped with emission spectral detection and a tunable laser providing excitation range from 470 to 670 nm. Stacks of optical sections were collected for computer-assisted neuron reconstructions.

Primary antibodies (Table 1) were detected with fluorophore-conjugated secondary antibodies for wide-field epifluorescence and confocal microscopy. After preincubation in 10% normal donkey serum (NDS) in PBST, sections were incubated with a mix of up to three primary antibodies simultaneously diluted in PBST with 1% NDS. The following secondary antibodies were used (all from Jackson Immunoresearch): donkey anti-chicken Alexa 488 (1:1000, 703-545-155), donkey anti-rat Cy3 (1:500, 712-165-150), donkey anti-sheep Dylight 647 (1:250-1:500, 713-605-147), donkey anti-rabbit Dylight 594 (1:500, 711-585-152), and donkey anti-rat Alexa 594 (1:500, 705-585-003).

**Table 1:** List of primary antibodies.

Molecule	Host	Dilution	Source	Antigen	RRID	Specificity information
GABA	Rabbit	1:1000	Sigma, A2052	GABA	AB_477652	Positive binding with GABA, and GABA-KLH in a dot blot assay, and negative binding with BSA
GFP	Chicken	1:1000	Aves Labs, GFP-1020	Recombinant GFP	AB_10000240	No staining observed in sections of GFP− brains from littermates.
GFP	Rabbit	1:15000	Invitrogen, A6455	Recombinant GFP	AB_221570	No staining observed in sections of GFP− brains from littermates.
Metabotropic glutamate receptor 2 (M2R)	Rat	1:500	Synaptic Systems, 223017	Recombinant protein of human M2R 207–388 aa	AB_2238208	No labeling in M2R knockout mice.
Neuronal nitric oxide synthase (nNOS)	Goat	1:500	Abcam, ab1376	Synthetic peptide: ESKKDTDEVFSS, corresponding to amino acids 1423–1434 of Human NOS1	AB_300614	Detects a band of ~160 kDa (predicted molecular weight: 161 kDa). Can be blocked with human nNOS peptide.
Neuropeptide Y (NPY)	Rabbit	1:5000	Immunostar, 22940	Native NPY	AB_2307354	All staining is blocked by pre-absorption of the diluted antiserum with excess NPY. Absorption with other peptides does not reduce the intensity of staining.
Parvalbumin (PV)	Goat	1:1000	Swant, pvg-214	Purified rat muscle PV	AB_10000345	No labeling in PV knockout mice
Somatostatin (SOM)	Rat	1:250	Millipore, MAB354	Synthetic peptide 1–14 aa	AB_2255365	No cross-reactivity to enkephalins, other endorphins, substance P or CGRP.
SOM	Goat	1:3000	Santa Cruz, sc-7819	Epitope mapping near the C-terminus of Somatostatin of human origin	AB_2302603	Band detected with Western blot in human Somatostatin-transfected 293 whole cell lysates but not in non-transfected ones.

For immunostainings with multiple antibodies, an initial negative control was performed by omitting each primary antibody in turn from the staining procedure; in these cases, no positive fluorescence signal was detected. In addition, each secondary antibody was omitted in turn to confirm its specificity. Epifluorescence images were obtained with a Zeiss AxioImager Z2 microscope coupled to a camera (Zeiss AxioCam MR3) with an HBO lamp associated with 470/40, 525/50, 545/25, and 605/70 filter cubes. Confocal images were acquired either with the Leica system described above or with a Zeiss LSM-800 system equipped with emission spectral detection and a tunable laser providing excitation range from 470 to 670 nm.

### Quantification of PV-expressing axon terminals

The innervation of ctrlGABA and ebGABA by PV + axon terminals was assessed in PFA-fixed sections stained for PV from GAD67-GFP and Dlx1/2(E7.5)-GFP mice, respectively. Confocal stacks centered on the soma and proximal dendrites of GFP + cells were acquired with a Zeiss LSM-800 microscope at constant resolution (0.065 µm/pixel) and z-step (0.41 µm). Since PV + axon density varies depending on CA1 layers, ctrlGABAs were sampled to match as much as possible the location of ebGABAs (ctrlGABAs: 17 from stratum oriens, 26 from intra-/peri-stratum pyramidale, 4 from stratum radiatum; ebGABA: 16 from stratum oriens, 14 from intra-/peri-stratum pyramidale, 2 from stratum radiatum). Appositions between PV + boutons and GFP + somata or proximal dendrites were counted manually using the cell counter plugin in Fiji (http://fiji.sc). Area and median fluorescence of the PV staining were quantified using first the threshold function to exclude unspecific signal.

### Neurolucida reconstruction and morphometric analysis

Fifty-two neurobiotin-filled neurons (38 filled in developing slices, 14 filled in adult slices) were reconstructed using Neurolucida (MBF Bioscience). Neurons recorded during development underwent morphometric analysis. Examined morphological variables included: dendritic and axonal lengths, dendritic, and axonal surfaces.

### Estimate of long-range projecting neurons originating from ebGABA

The proportion of ebGABAs formed by long-range projecting cell types was estimated as follows. We summed: (1) the percentage of ebGABAs formed by SOM+ (but nNOS−) cells in stratum oriens; (2) the percentage of ebGABAs formed by strong nNOS+ cells; (3) the percentage of ebGABAs formed by M2R+ (but nNOS−) cells.

SOM+ cells in stratum oriens are very likely to be projection cells for three reasons. First, most of them are not O-LM cells (very few express PV and we never observed a dense axonal plexus in lacunosum-moleculare in filled cells). Second, most of them are not bistratified cells because very few ebGABAs co-express NPY and PV^55^. Third, the majority of septum-projecting CA1 GABA cells are ebGABAs with the soma in the stratum oriens, and ∼80% of them express SOM^12^.

### Drugs

NBQX disodium salt and SR95531 (gabazine) were purchased from Tocris Biosciences. All the remaining drugs (tamoxifen and compounds to prepare ACSF and intracellular solutions) were purchased from Sigma.

### Statistical analyses

Pairwise comparisons between distributions were performed using the Mann–Whitney *U* test for unpaired groups and with the Wilcoxon signed rank test for paired groups. Comparisons between multiple groups were performed using the Friedman test with post hoc Dunn’s correction. Two-way ANOVAs were performed with Bonferroni *post hoc* correction. Data are expressed as either means ± SDs or medians and IQR. Statistical tests were performed with Graphpad Prism 8 (GraphPad Software, Inc.) or MATLAB.

### Reporting summary

Further information on research design is available in the Nature Research Reporting Summary linked to this article.

## Supplementary information

Supplementary Information

Peer Review File

Supplementary Movie 1

Supplementary Movie 2

Supplementary Movie 3

Reporting Summary

## Data Availability

In vivo two-photon imaging data are available at 10.5281/zenodo.3931805. The remaining raw data are available from the authors upon request. Source data are provided with this paper.

## Source data

Source Data

## Code availability

MATLAB codes to analyze in vitro and in vivo two-photon imaging data as well as intrinsic membrane properties are available at https://gitlab.com/cossartlab/bocchio-gouny-et-al-2020. The code for in vivo functional connectivity analysis is part of our CICADA Python toolbox, available at https://gitlab.com/cossartlab/cicada/.
