# Supplementary material for: Hippocampal hub neurons maintain distinct connectivity throughout their lifetime
Source: Nat Commun. 2020 Sep 11;11:4559. doi: 10.1038/s41467-020-18432-6 (PMC7486410; doi:10.1038/s41467-020-18432-6)
Supplement: Supplementary file 1 — Supplementary Information [file 41467_2020_18432_MOESM1_ESM.pdf]

Hippocampal hub neurons maintain distinct connectivity throughout their lifetime

Marco Bocchio*, Claire Gouny*, David Angulo-Garcia, Tom Toulat, Thomas Tressard, Eleonora Quiroli, Agnès Baude & Rosa Cossart

Supplementary information

Contains:

- 2 Supplementary Tables
- 9 Supplementary Figures

Supplementary table 1 (related to Figure 2)
Effect of stimulation of ctrlGABAs and ebGABAs on GDP occurrence

GABA cells (#)	Stimulation frequencies (Hz)	GDP rate	GDP synchronization	
		Effect and *P*-value	*P*-value	Delay of evoked GDP after stimulation (s)
ebGABA #d16	0,1	ns	< 0.001	1.2
	0,2	ns	< 0.05	0.4
	0,4	ns	< 0.01	1.93
ebGABA #d20	0,1	ns	< 0.05	3.2
	0,2	↓ < 0.01	ns	na
	0,4	ns	< 0.001	0.16
ebGABA #d29	0,1	ns	ns	na
	0,2	↑ < 0.05	ns	na
	0,4	ns	< 0.05	0.96
ebGABA #d33	0,2	ns	< 0.01	3.6
	0,4	↓ < 0.01	< 0.05	2.16
ebGABA #d39	0,1	ns	ns	na
	0,2	ns	< 0.05	3.76
	0,4	ns	< 0.05	2.16
ebGABA #d53	0,1	ns	< 0.05	5.84
	0,2	ns	< 0.001	2.48
	0,4	ns	< 0.01	0.96
ctrlGABA #d66	0,2	ns	< 0.05	3.03
	0,4	ns	ns	na
ctrlGABA #d67	0,2	ns	ns	na
	0,4	ns	ns	na
ctrlGABA #d68	0,2	ns	ns	na
	0,4	ns	ns	na
ctrlGABA #d69	0,2	ns	ns	na
	0,4	ns	ns	na
ctrlGABA #d70	0,2	ns	ns	na
	0,4	ns	ns	na
ctrlGABA #d75	0,2	ns	ns	na
	0,4	ns	ns	na
ctrlGABA #d80	0,2	ns	ns	na
	0,4	ns	ns	na
ctrlGABA #d82	0,2	ns	ns	na
	0,4	ns	ns	na

Upward pointing arrow means significant increase in GDP rate; downward pointing arrows mean decrease in GDP rate. *P* values for differences in GDP rate were calculated using two-sided Mann-Whitney U tests. *P* values for GDP synchronization were calculated using two-sided permutation tests (see Methods).

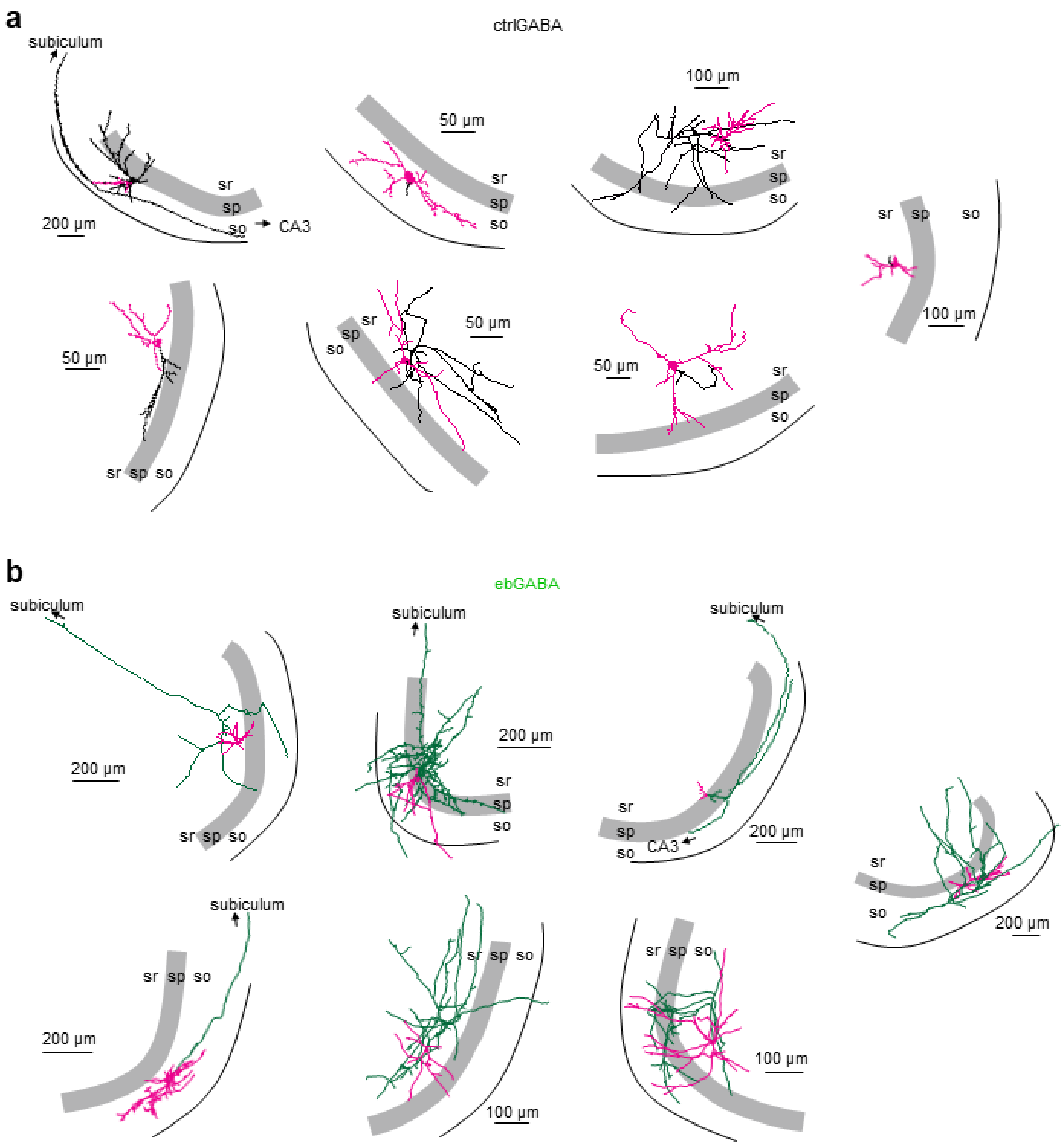

Supplementary figure 1 (related to Figure 2)

Axonal and dendritic fields of ctrlGABAs and ebGABAs of the developing CA1

Neurolucida reconstructions of seven ctrlGABA (panel a) and seven ebGABA cells (panel b). Axon is depicted in green for ebGABA and in black for ctrlGABA. Soma and dendrites are colored in magenta. so: stratum oriens; sp: stratum pyramidale; sr: stratum radiatum.

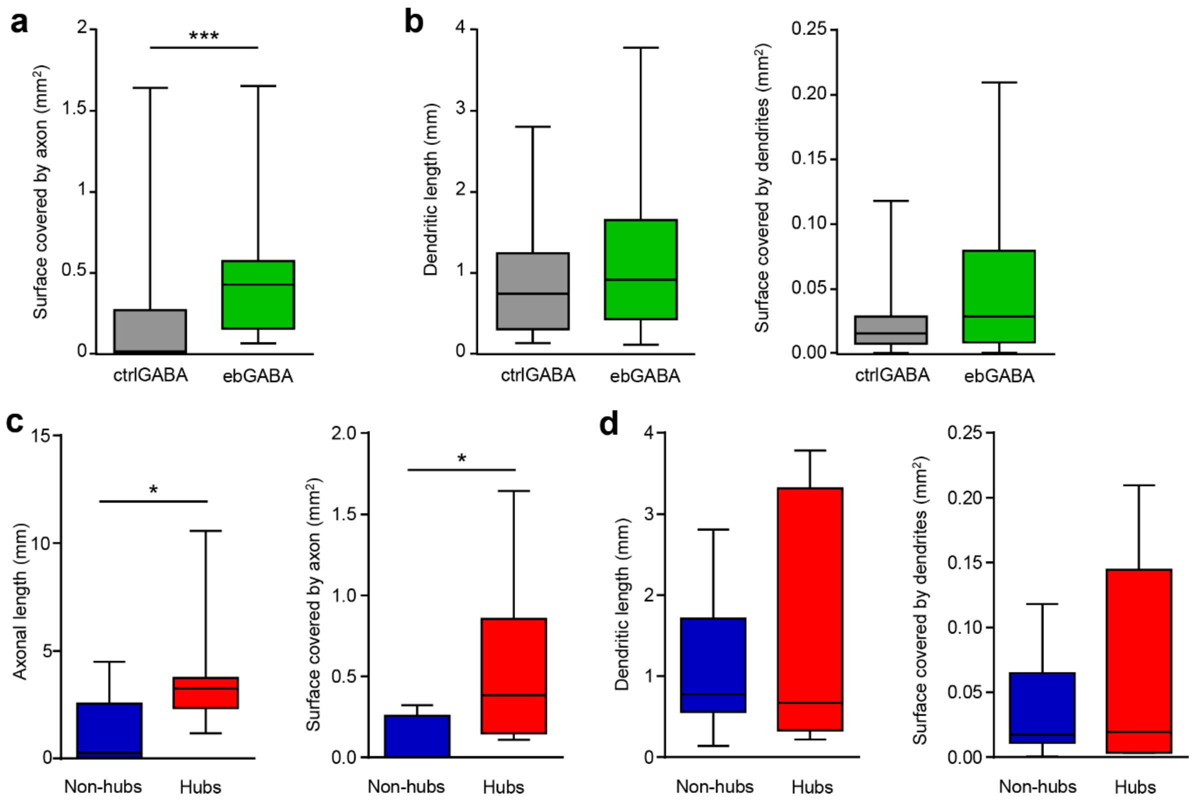

Supplementary figure 2 (related to Figure 2)

Anatomical features of hub cells in the developing CA1

(a) The axons of ebGABAs (n=18 cells) cover a significantly bigger surface than the axons of ctrlGABAs (n=20 cells, *P*=0.0003, two-sided Mann-Whitney U test). **(b)** Dendritic length and surface covered by dendrites do not differ significantly between ctrlGABAs and ebGABAs (*P*=0.279 and *P*=0.125, respectively, n=20 ctrlGABAs, n=18 ebGABAs, two-sided Mann-Whitney U test). **(c-d)** Cells exerting a hub function in the network (affecting either GDP frequency or GDP synchronization, n=7 cells) display significantly longer axons *(c)* but not dendrites *(d)* than non-hub cells (n=7 cells, axonal length *P*=0.0379, surface covered by axon *P*=0.0175, dendritic length *P*=0.872, surface covered by dendrites *P*=0.779, two-sided Mann-Whitney U test). Data are represented as medians (interquartile ranges). Boxplot whiskers represent minimum and maximum values.

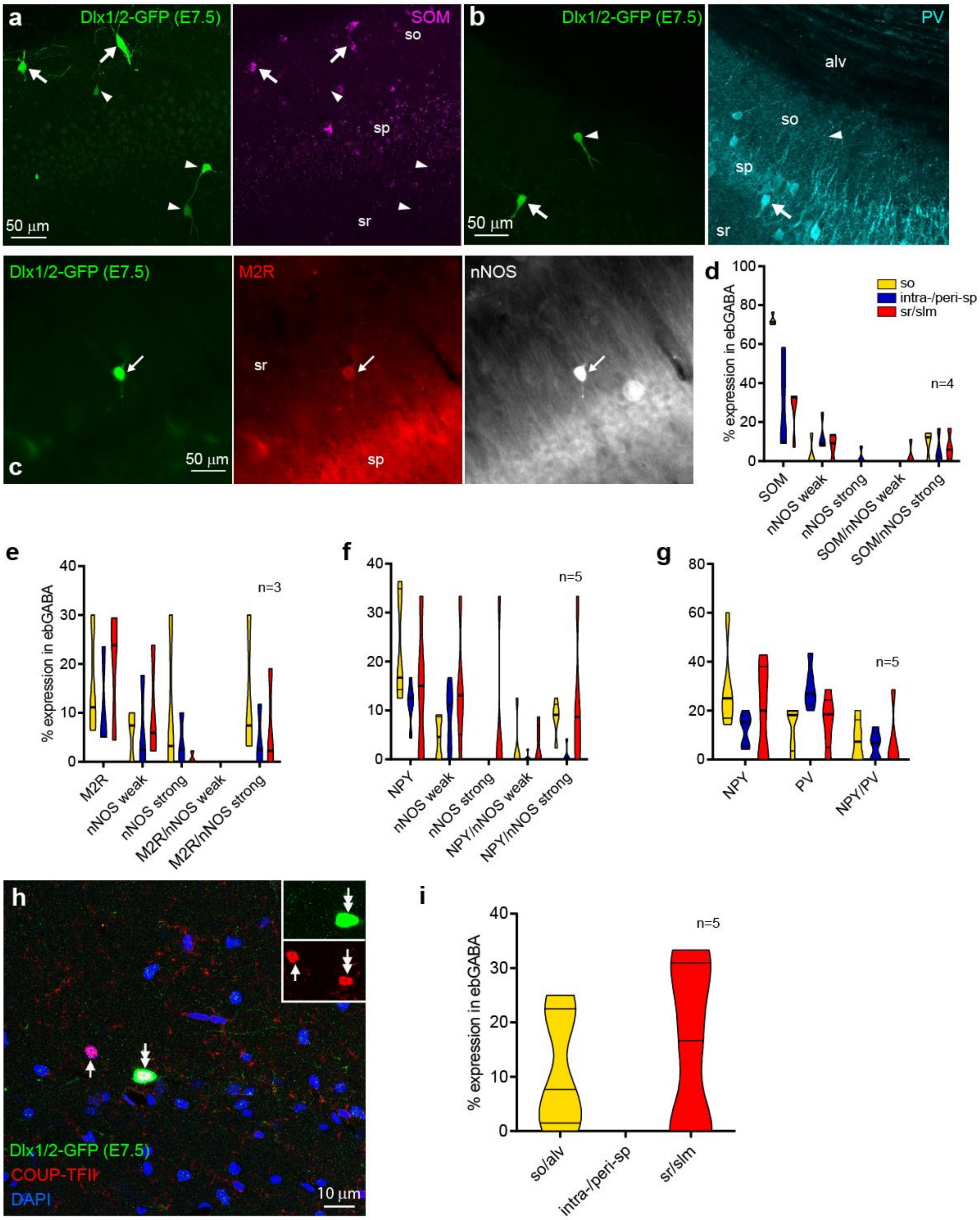

Supplementary figure 3 (related to Figure 3)

Neurochemical markers expressed by ebGABA in the adult CA1

(a) Representative ebGABA cells immunopositive (arrows) or immunonegative (arrowheads) for SOM. Note the higher expression of SOM in stratum oriens. Experiment repeated independently in four mice with similar results. **(b)** One representative ebGABA at the border between the stratum pyramidale and the stratum radiatum expresses PV (arrow), while another ebGABA in stratum oriens is PV immunonegative (arrowhead). Experiment repeated independently in five mice with similar results. **(c)** EbGABA in stratum radiatum expressing both M2R and strong levels of nNOS (arrow). Experiment repeated independently in three mice with similar results. **(d-g)** Quantification of the co-expression of two molecular markers in ebGABA. Proportional expression refers to each layer (and not to the whole CA1). **(h)** Two representative COUP-TFII+ cells: one immunopositive ebGABA (double arrow) and a GFP-negative cell (single arrow). Experiment repeated independently in five mice with similar results. **(i)** Quantification of the expression of COUP-TFII in ebGABAs. Proportional expression refers to each layer (and not to the whole CA1). Lines in violin plots show quartiles. The number of mice from which the data were obtained are indicated next to the

violin plots. so**:** stratum oriens; sp: stratum pyramidale; sr: stratum radiatum; slm: stratum lacunosum-moleculare.

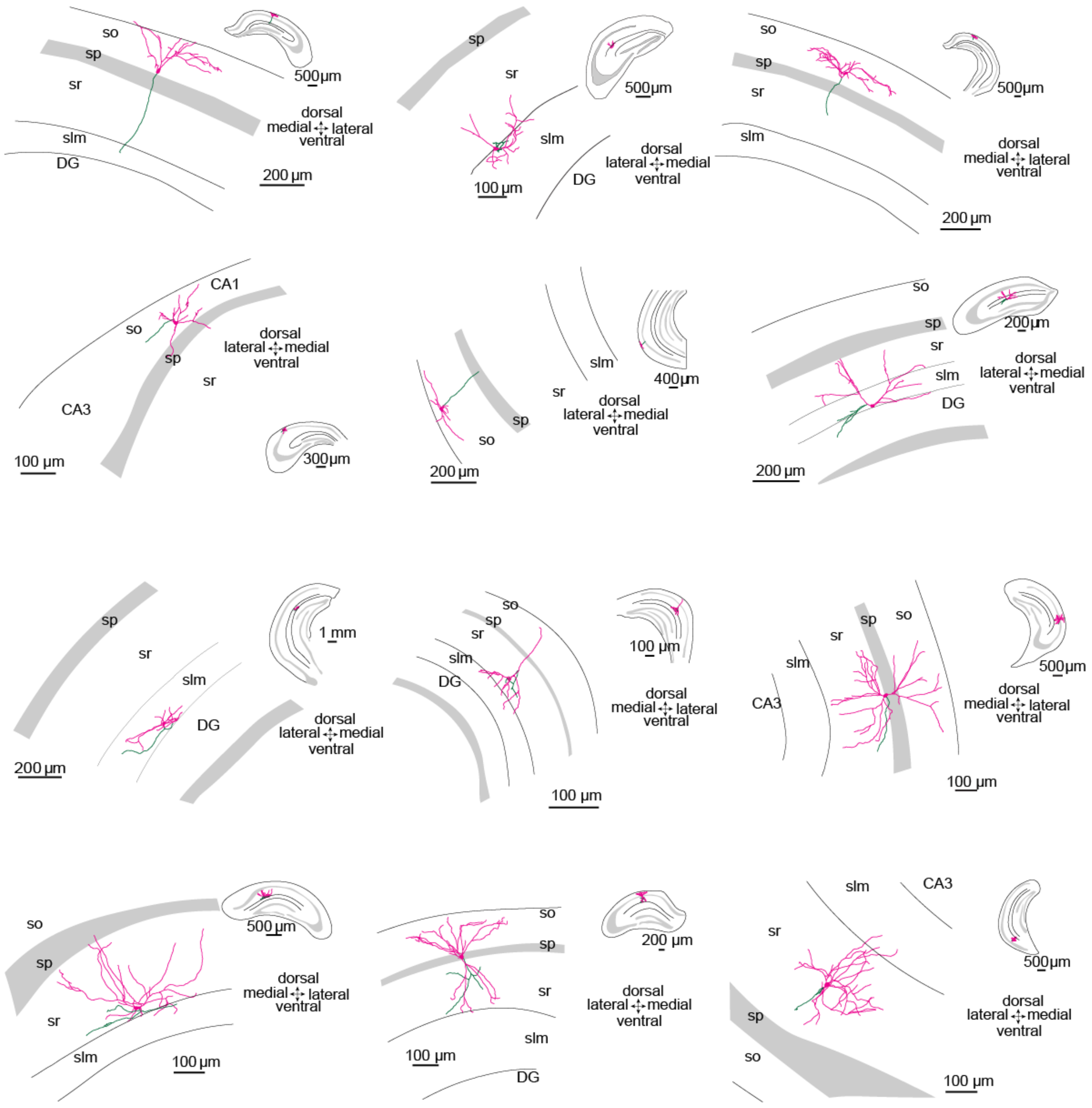

Supplementary figure 4 (related to Figure 3)

Axonal and dendritic fields of ebGABAs in the adult CA1

Neurolucida reconstructions of neurobiotin-filled ebGABA in the adult CA1. Axon is depicted in green for ebGABA. Soma and dendrites are colored in magenta. Inset: zoomed out representation showing the entire hippocampus to illustrate the rostro-caudal and dorso-ventral position of the neurons. so: stratum oriens; sp: stratum pyramidale; sr: stratum radiatum; slm: stratum lacunosum-moleculare.

Supplementary table 2 (related to Figure 3)

Intrinsic electrophysiological properties of ctrlGABAs and ebGABAs in the adult CA1

**Parameter**	**ctrlGABA n=18**	**ebGABA n=16**	***P* value**
Rheobase (pA)	49 (27 – 164)	39 (23 – 82)	0.165
V_m rest (mV)	-80.0 (-83.0 – -76.8)	-75.2 (-82.1 – -70.2)	0.125
R_{in} (MΩ)	320 (123 – 414)	357 (256 – 561)	0.327
Membrane τ (ms)	44.4 (33.4 – 77.4)	36.7 (28.6 – 46.1)	0.108
Capacitance (pF)	114.4 (88.3 – 674.7)	89.3 (56.2 – 180.8)	0.185
Spike threshold (mV)	-42.5 (-44.9 – -38.3)	-42.2 (-45.0 – -35.1)	0.437
Spike amplitude (mV)	63.5 (46.3 – 69.7)	61.1 (56.8 – 66.6)	0.952
Spike half-width (ms)	0.7 (0.5 – 0.9)	0.8 (0.6 – 1.1)	0.198
AHP_{fast} (mV)	11.0 (8.7 – 15.7)	14.1 (11.2 – 14.9)	0.132
Sag ratio	0.472 (0.378 – 0.557)	0.590 (0.439 –0.619)	0.028
Firing rate (2× rheobase, Hz)	21 (13– 74)	18 (14 – 25)	0.369
Maximum firing rate (Hz)	123 (60 – 201)	74 (55 – 103)	0.047
Adaptation index (2× rheobase)	0.661 (0.552 – 0.746)	0.741 (0.660 – 0.767)	0.551
Burst index (2× rheobase)	0.069 (0.063 – 0.091)	0.068 (0.056 – 0.077)	0.449
CV ISI (2× rheobase)	0.163 (0.131 – 0.266)	0.181 (0.109 – 0.249)	0.852

Values are expressed as medians (inter-quartile range). Distributions were compared with a Mann-Whitney U test. Significantly different distributions are highlighted in red. V_m rest: resting membrane potential; R_{in}: input resistance; AHP_{fast}: fast afterhyperpolarization; CV ISI: coefficient of variation of the inter-spike interval. Red color denotes significantly different parameters

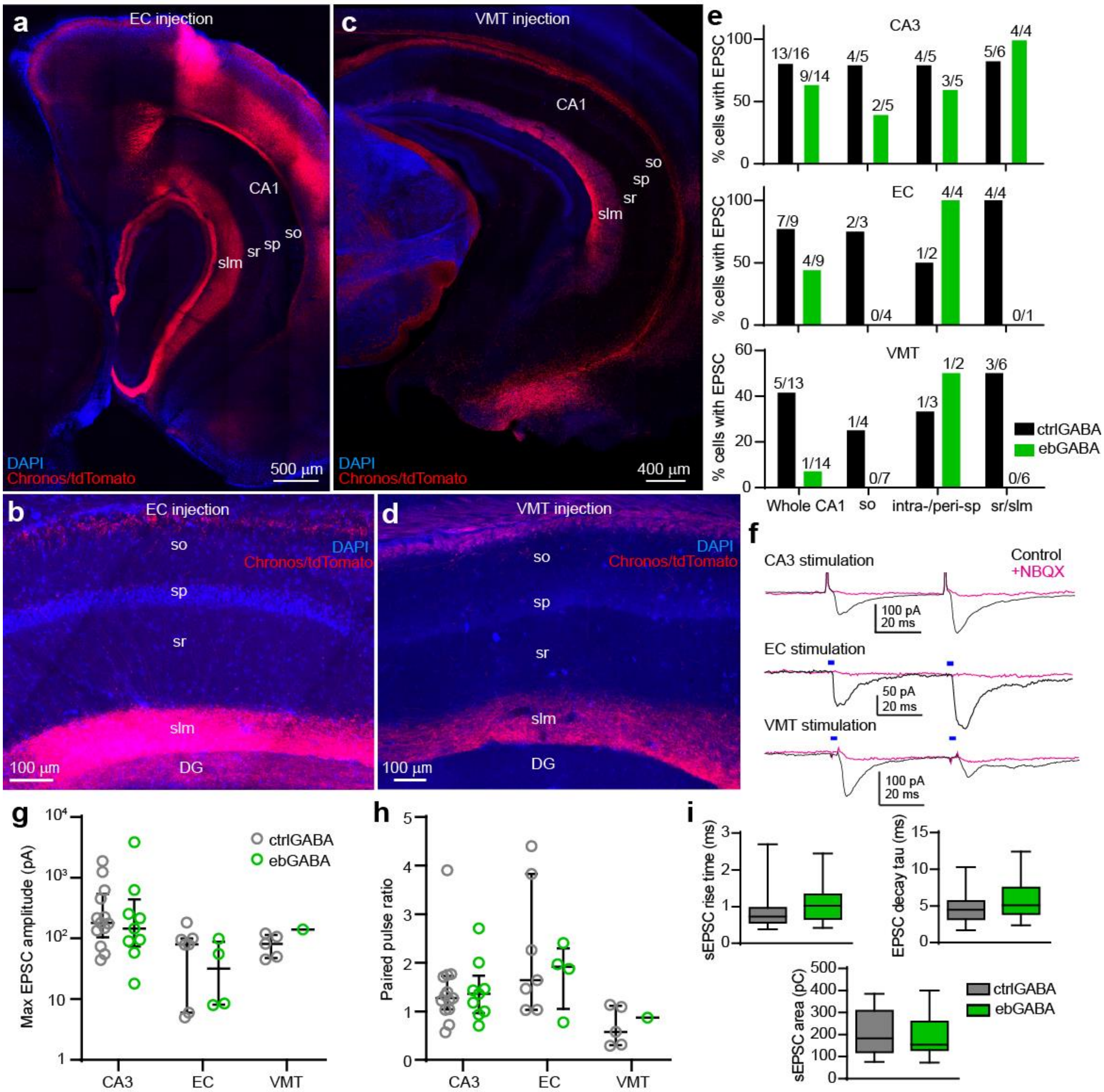

Supplementary figure 5 (related to Figure 4)

Properties of excitatory inputs to CA1 GABA cells

(a) Axons from the entorhinal cortex (EC) innervating the hippocampus. Note that afferents innervating CA1 are densest in the stratum lacunosum-moleculare. Experiment repeated in 15 mice with similar result **(b)** Zoomed-in view of the entorhinal projection to CA1. **(c)** Axons from the ventromedial thalamus innervating the hippocampus. Note that afferents are restricted to CA1 and, within this region, they are densest in the stratum lacunosum-moleculare. Experiment repeated in seven mice with similar result **(d)** Zoomed-in view of the thalamic projection to CA1. **(e)** Three example EPSCs evoked by stimulation of CA3, entorhinal and thalamic axons. All EPSCs are glutamatergic because they are abolished (or greatly reduced) by NBQX (10 µM). **(f-g)** Similar maximum amplitude and paired pulse ratio of EPSCs evoked in ctrlGABAs and ebGABAs from stimulation of CA3 (max amplitude *P*=0.675, PPR *P*=0.988, two-sided Mann-Whitney U test, n=13 CtrlGABAs, n=9 ebGABAs), entorhinal (max amplitude *P*=0.588, PPR *P*=0.842, two-sided Mann-Whitney U test, n=7 CtrlGABAs, n=4 ebGABAs) or thalamic afferents (no statistics given low number of responses, n=5 CtrlGABAs, n=1 ebGABAs). Only responsive cells were included in this quantification. **(h)** Similar sEPSC kinetics in ctrlGABAs and ebGABAs (rise time *P*=0.15, decay tau: *P*=0.8, area *P*=0.477, two-sided Mann-

Whitney U tests, n=18 cells for both groups). so: stratum oriens; sp: stratum pyramidale; sr: stratum radiatum; slm: stratum lacunosum-moleculare; DG: dentate gyrus. Data are represented as medians (interquartile ranges). Boxplot whiskers represent minimum and maximum values.

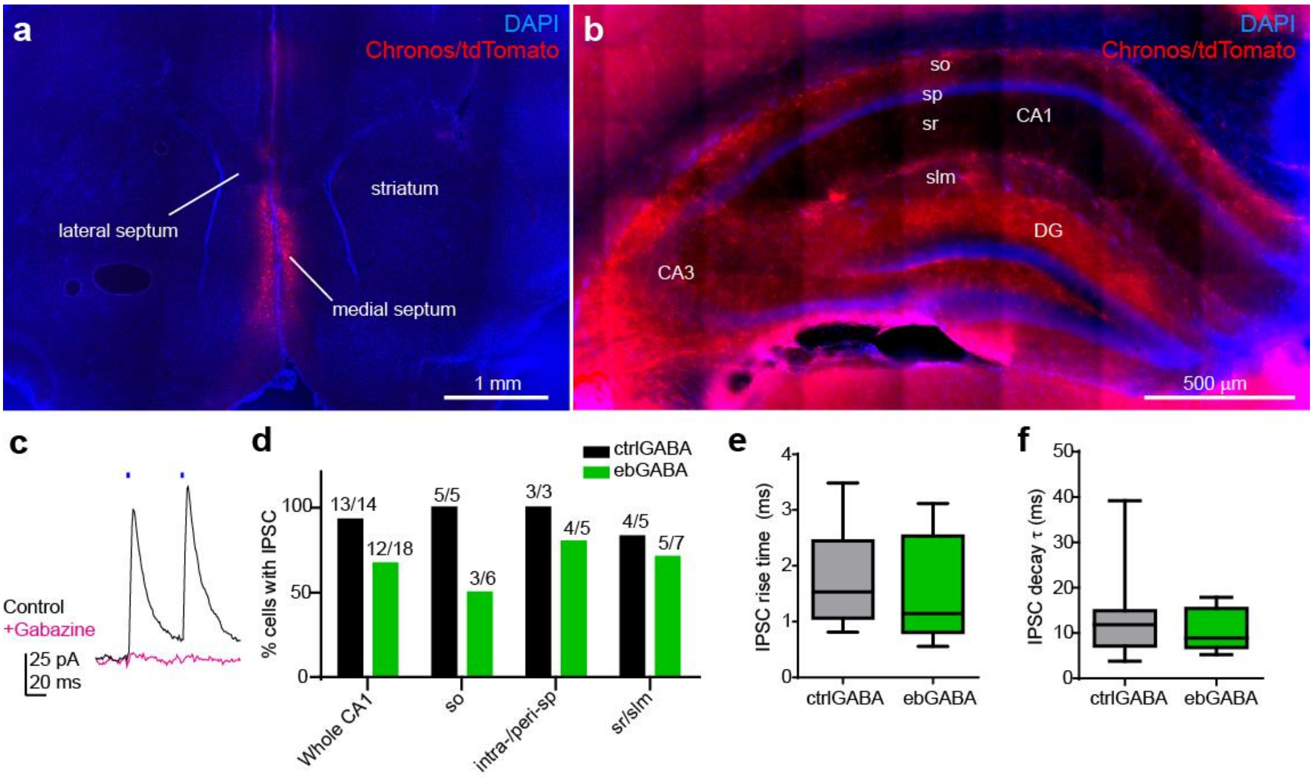

Supplementary figure 6 (related to Figure 5)

Properties of inhibitory inputs onto CA1 GABA cells

(a) expression of Chronos/tdTomato in the medial septum of a representative mouse. Experiment repeated in ten mice with similar results. **(b)** Axons from medial septal neurons innervating the hippocampus. Note that within CA1 afferents innervate all layers but are particularly dense in stratum oriens and at the border between stratum radiatum and stratum lacunosum moleculare. Experiment repeated in ten mice with similar results. **(c)** IPSC evoked by optical stimulation of medial septal afferents is blocked by gabazine (SR95531, 10 µM). **(d)** Proportion of CA1 ctrlGABA and ebGABA cells receiving GABAergic inputs from the medial septum. No significant difference in the proportions for the whole CA1 (P=0.104, two-sided Fisher's exact test, n=14 ctrlGABAs, n=18 ebGABAs). **(e-f)** Similar septal IPSC rise time (P=0.43, two-sided Mann Whitney U test) and decay time (P=0.683, two-sided Mann Whitney U tests) in ctrlGABAs (n=13 cells) and ebGABAs (n=12 cells). so: stratum oriens; sp: stratum pyramidale; sr: stratum radiatum; slm: stratum lacunosum-moleculare; DG: dentate gyrus. Distributions are represented as medians (interquartile ranges). Boxplot whiskers represent minimum and maximum values.

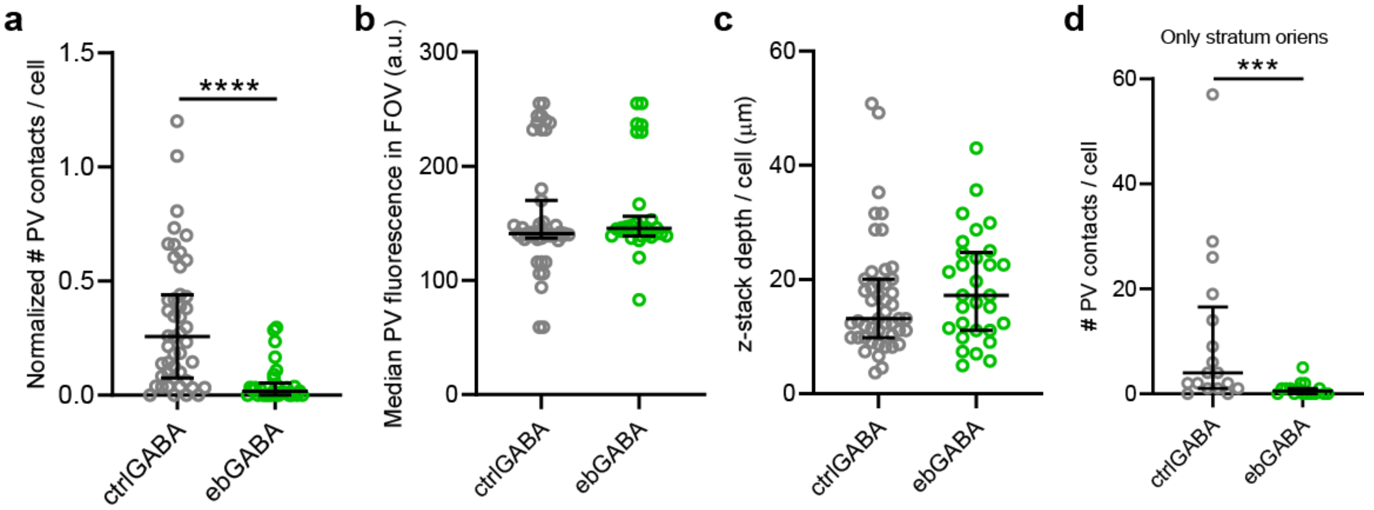

Supplementary figure 7 (related to Figure 5)

Low amount of PV contacts onto ebGABAs is not due to uneven sampling or staining quality

(a) Difference in the amount of PV+ boutons between ctrlGABAs (n=47 cells) and ebGABAs (n=30 cells) persists even when the number of contacts is normalized by the number of optical sections scanned for each cell (*P*<0.0001, two-sided Mann-Whitney U test). **(b)** Similar median fluorescence of the PV staining for examined ctrlGABAs and ebGABAs cells (*P*=0.275, two-sided Mann-Whitney U test, n=47 ctrlGABAs, n=30 ebGABAs). **(c)** No difference in the z-stack depth for imaged ctrlGABAs and ebGABAs (*P*=0.23, two-sided Mann-Whitney U test, n=47 ctrlGABAs, n=30 ebGABAs). **(d)** Difference in the amount of PV+ boutons between ctrlGABAs and ebGABAs persists even when analysis is restricted to cells with the soma in the stratum oriens (*P*=0.0008, two-sided Mann-Whitney U test, n=47 ctrlGABAs, n=30 ebGABAs). Data are represented as scatter plots with medians and interquartile ranges. *** *P*<0.001. **** *P*<0.0001.

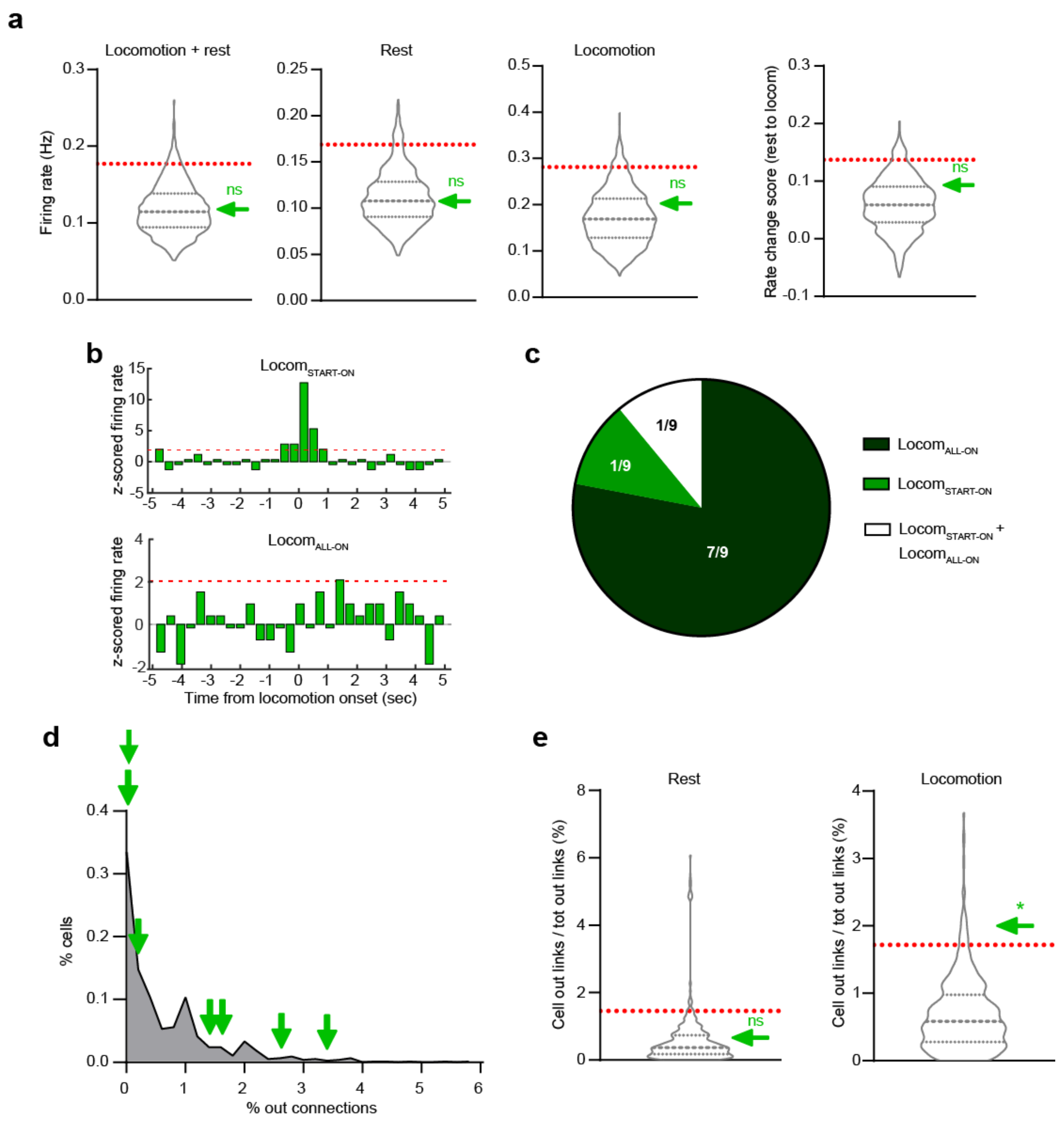

Supplementary figure 8 (related to Figure 6)

Activity of ebGABAs in the adult CA1 *in vivo*

(a) Mean inferred firing rates of ebGABAs (n=9 cells from seven FOVs) in relation to surrogate distributions of control cells (n=776, matched subsampling of GFP-negative cells for each FOV repeated 1000 times, violin plots). The mean firing rate of ebGABAs is not significantly different from the mean firing rate of control neurons. No significant difference could be detected in the entire recording period or when restricting the analysis to periods of locomotion or rest (all P>0.05, two-sided bootstrap). Rate change scores did not differ between ebGABAs and controls (P>0.05, two-sided bootstrap). The rate change score is defined as: $(rate_{LOCOM} - rate_{REST}) / (rate_{LOCOM} + rate_{REST})$. **(b)** Z-scored PSTHs showing one ebGABA significantly activated at locomotion onset (Locom$_{START\text{-}ON}$, *top*) and one that fired significantly more during locomotion than rest (Locom$_{ALL\text{-}ON}$, see Methods) but showed no activation at locomotion onset (*bottom*). Red dashed lines depict statistical threshold. Cells were defined as significantly activated if at least two consecutive bins exceeded a Z-score of 2 in a time window from 200 ms before locomotion onset onwards. **(c)** Proportions of responses to locomotion displayed by ebGABAs. The majority of ebGABAs were broadly more active during locomotion than rest, but not at locomotion onset (Locom$_{ALL\text{-}ON}$). One cell showed activation only at locomotion onset (Locom$_{START\text{-}ON}$). Another showed activation at both locomotion onset and throughout the entire locomotion

periods compared to rest ($Locom_{START-ON}$ + $Locom_{ALL-ON}$). **(d)** Histogram of the percentage of output connections (over the total amount of connections in each FOV) for all FOVs in the stratum pyramidale pooled (n=5 FOVs). Green arrows mark bins containing ebGABAs (n=7 cells). EbGABAs display heterogeneous functional output connectivity. **(e)** Percentage of functional output connections over the total amount of connections in the FOV for rest and locomotion periods. EbGABAs' (n=7 cells from seven FOVs) average percentages (green arrows) were compared to surrogate distributions of control cells (matched subsampling of GFP-negative cells for each FOV repeated 1000 times, violin plots). EbGABAs display a higher proportion of output connections than control cells (n=746) during locomotion (P<0.05, two-sided bootstrap) but not during rest (P>0.05, two-sided bootstrap). Red dashed lines depict statistical thresholds (see Methods for details). Dashed lines in violin plots show medians and quartiles. * *P*<0.05.

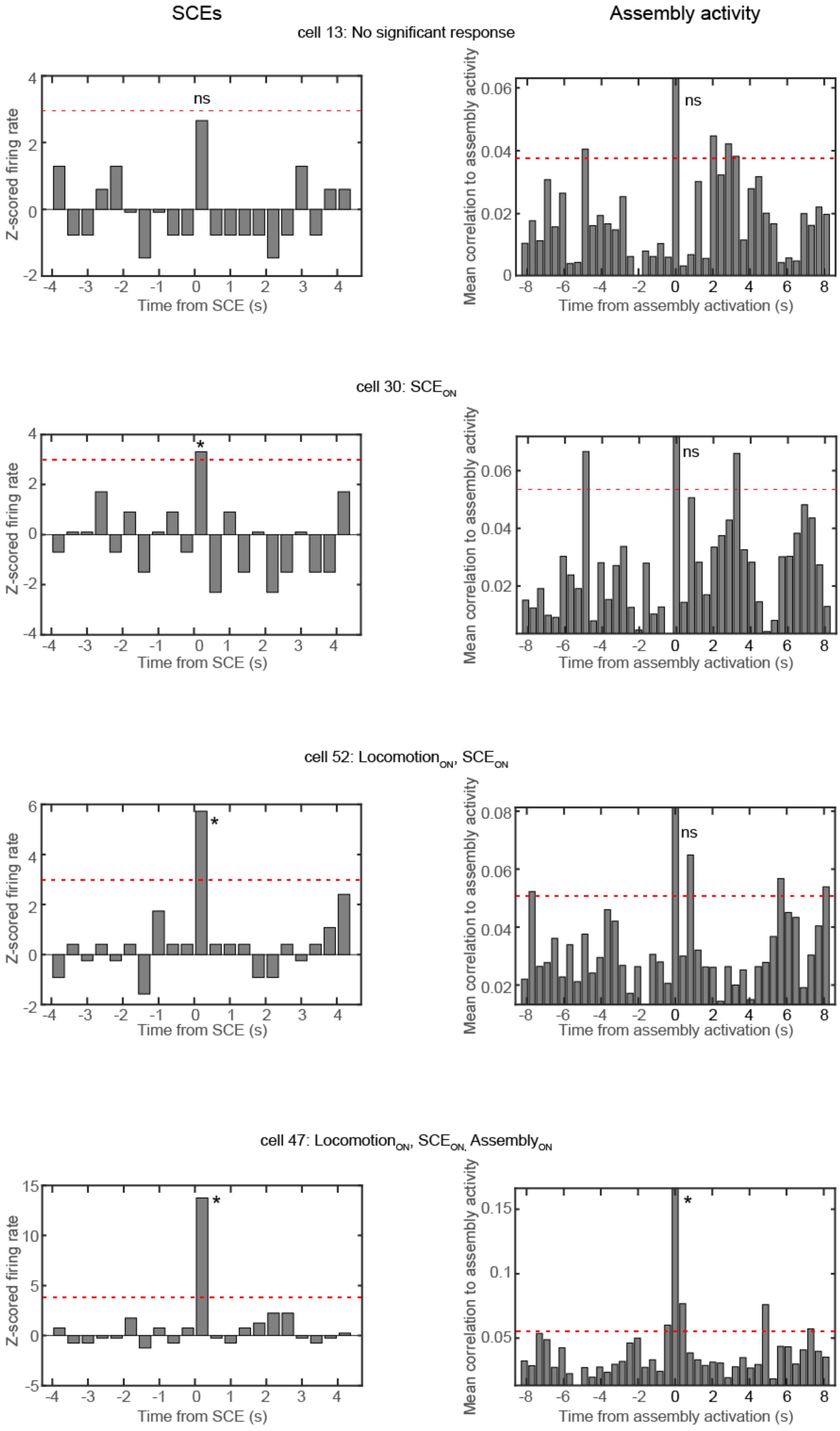

Supplementary figure 9 (related to Figure 6)

Diversity of single cell responses to locomotion and network events in the adult CA1

Activity of four cells in relation to SCEs (left) and assembly activity (right). Red dashed lines depict statistical thresholds (see Methods for details).
