# Supplementary material for: Hippocampal hub neurons maintain distinct connectivity throughout their lifetime
Source: Nat Commun. 2020 Sep 11;11:4559. doi: 10.1038/s41467-020-18432-6 (PMC7486410; doi:10.1038/s41467-020-18432-6)
Supplement: Supplementary file 6 — Reporting Summary [file 41467_2020_18432_MOESM6_ESM.pdf]

nature research

Corresponding author(s): Rosa Cossart

Last updated by author(s): 25/11/19

Reporting Summary

Nature Research wishes to improve the reproducibility of the work that we publish. This form provides structure for consistency and transparency in reporting. For further information on Nature Research policies, see Authors & Referees and the Editorial Policy Checklist.

Statistics

For all statistical analyses, confirm that the following items are present in the figure legend, table legend, main text, or Methods section.

n/a	Confirmed	
☐	☒	The exact sample size (*n*) for each experimental group/condition, given as a discrete number and unit of measurement
☐	☒	A statement on whether measurements were taken from distinct samples or whether the same sample was measured repeatedly
☐	☒	The statistical test(s) used AND whether they are one- or two-sided<br>*Only common tests should be described solely by name; describe more complex techniques in the Methods section.*
☐	☒	A description of all covariates tested
☐	☒	A description of any assumptions or corrections, such as tests of normality and adjustment for multiple comparisons
☐	☒	A full description of the statistical parameters including central tendency (e.g. means) or other basic estimates (e.g. regression coefficient) AND variation (e.g. standard deviation) or associated estimates of uncertainty (e.g. confidence intervals)
☐	☒	For null hypothesis testing, the test statistic (e.g. *F*, *t*, *r*) with confidence intervals, effect sizes, degrees of freedom and *P* value noted<br>*Give P values as exact values whenever suitable.*
☒	☐	For Bayesian analysis, information on the choice of priors and Markov chain Monte Carlo settings
☒	☐	For hierarchical and complex designs, identification of the appropriate level for tests and full reporting of outcomes
☒	☐	Estimates of effect sizes (e.g. Cohen's *d*, Pearson's *r*), indicating how they were calculated

Our web collection on statistics for biologists contains articles on many of the points above.

Software and code

Policy information about availability of computer code

Data collection	Calcium imaging data were acquired using ImSpector (LaVision Biotec, version 4.1). pClamp (Molecular Devices, version 10) was used to acquire imaging, electrophysiological and/or locomotion triggers and synchronize imaging data to whole cell current clamp data or locomotor activity. Patch clamp data were acquired using pClamp (Molecular Devices, version 10). Confocal imaging data were acquired using ZEN (Zeiss, version 2) or LAS AF (Leica Microsystems, version 2.7.3). Epifluorescence imaging data were acquired using ZEN (Zeiss, version 2).
Data analysis	Patch clamp data were analyzed using Clampfit (pClamp package, Molecular Devices, version 10), Mini Analysis (Synaptosoft, version 6.0.7) and custom codes written in MATLAB (Mathworks, version R2017b) available at https://gitlab.com/cossartlab/bocchio-gouny-et-al-2020. Motion correction, segmentation and spike inference of in vivo imaging data were performed using the NoRMCorre toolbox (https://github.com/flatironinstitute/NoRMCorre) and the CaImAn toolbox (https://github.com/flatironinstitute/CaImAn-MATLAB) for MATLAB (version R2017b). In vitro and in vivo imaging data were analyzed using custom codes written in MATLAB (version R2011a and R2017b, respectively) available at https://gitlab.com/cossartlab/bocchio-gouny-et-al-2020. The code for in vivo functional connectivity analysis is part of our CICADA Python toolbox, available at https://gitlab.com/cossartlab/cicada. Quantifications of axonal boutons and overlap between somatic markers were performed using the Fiji package for ImageJ (version 1.51). Neuronal reconstructions were carried out with Neurolucida (MBF, Bioscience, version 10). Statistical analyses were performed with GraphPad Prism (version 8.3) or MATLAB (version R2011a or R2017b).

For manuscripts utilizing custom algorithms or software that are central to the research but not yet described in published literature, software must be made available to editors/reviewers. We strongly encourage code deposition in a community repository (e.g. GitHub). See the Nature Research guidelines for submitting code & software for further information.

Data

Policy information about availability of data

All manuscripts must include a data availability statement. This statement should provide the following information, where applicable:

- Accession codes, unique identifiers, or web links for publicly available datasets
- A list of figures that have associated raw data
- A description of any restrictions on data availability

In vivo 2-photon imaging data are available at https://doi.org/10.5281/zenodo.3931805. The remaining raw data are available from the authors upon request. Source data are provided with this paper.

Field-specific reporting

Please select the one below that is the best fit for your research. If you are not sure, read the appropriate sections before making your selection.

☒ Life sciences ☐ Behavioural & social sciences ☐ Ecological, evolutionary & environmental sciences

For a reference copy of the document with all sections, see nature.com/documents/nr-reporting-summary-flat.pdf

Life sciences study design

All studies must disclose on these points even when the disclosure is negative.

Sample size

For imaging experiments, we chose a sample size between six and nine ebGABAs (six for in vitro imaging, nine for in vivo imaging experiments). These sample sizes allowed to perform meaningful statistics, while at the same time minimizing the number of animals used given that ebGABAs tagged with GFP are very sparse. This sample size is comparable to the sample size that was chosen in a recent publication for imaging a similarly sparse neuronal population (Francavilla, R., Villette, V., Luo, X. et al. Connectivity and network state-dependent recruitment of long-range VIP-GABAergic neurons in the mouse hippocampus. Nat Commun 9, 5043 (2018). https://doi.org/10.1038/s41467-018-07162-5).

For quantifications of neurochemical markers expressed by ebGABAs, we chose a sample of size of at least three animals. For patch clamp experiments and quantification of PV+ boutons, we chose sample sizes above 14 cells per groups (range: 14-43 cells) because this allowed to both sample evenly across CA1 layers and perform meaningful statistical comparisons. These sample sizes are comparable to the ones chosen for similar experiments in other studies in the field (for instance, Chittajallu R, Craig MT, McFarland A, et al. Dual origins of functionally distinct O-LM interneurons revealed by differential 5-HT(3A)R expression. Nat Neurosci. 2013;16(11):1598-1607. doi:10.1038/nn.3538).

Data exclusions

For in vitro electrophysiology, cells with initial series resistance > 30 MΩ or cells in which series resistance changed by more than 20% from the initial value were excluded from the analysis. For in vitro imaging, movies in which no giant depolarizing potentials could be detected in baseline conditions were omitted from subsequent analyses. For in vivo imaging data, analyses of the calcium dynamics during spontaneous locomotion and rest could be performed only for nine ebGABAs and nearby cells (n = 776 in total) from seven FOVs from six mice (two FOVs from stratum oriens and six from the stratum pyramidale). The remaining eight ebGABAs could not be analyzed because of either excessive movement in the z-axis (four cells), no expression of jRGECO1a in the Dlx1/2(E7.5)-GFP+ cells (three cells) or epileptic-like activity detected in the FOV (one cell). Exclusion criteria are standard in the field and were pre-established.

Replication

Experiments to obtain data presented in Fig. 2h-i, 3a-c, 6 and Supplementary Fig. 1-3 were run by two different experimenters that obtained similar results. The remaining experiments were performed by only one experimenter. However, reproducibility was ensured by sampling from several animals (at least three for anatomical quantifications, at least six for neurophysiology experiments) and verifying that results obtained from different animals were comparable. The finding that ebGABAs receive weak local inhibition was cross-checked with three separate experiments (Fig. 5 and Supplementary Fig. 7).

Randomization

Mice were not allocated to different experimental groups. Mice were selected randomly from group-housed cages. CtrlGABAs consist of randomly targeted putative GABAergic cells (electrophysiology experiments) or GAD67-expressing cells (Fig. 5i-j and Supplementary Fig. 7). In order to avoid biases towards specific interneuron types or innervation from specific pathways, ctrlGABAs and ebGABAs were sampled in even proportions from different layers (approx. one third from stratum oriens, one third from stratum pyramidale, one third from strata radiatum and lacunosum-moleculare). Since membrane and synaptic parameters may be affected by age, for all adult datasets we tested that the ages of the mice from which ctrlGABAs and ebGABAs were sampled did not differ significantly. Additionally, for optogenetics experiments we verified that the time of opsin expression did not differ between the two groups.

Blinding

Blinding was not possible because all experiments required targeting GFP-expressing or GFP-negative cells.

Reporting for specific materials, systems and methods

We require information from authors about some types of materials, experimental systems and methods used in many studies. Here, indicate whether each material, system or method listed is relevant to your study. If you are not sure if a list item applies to your research, read the appropriate section before selecting a response.

Materials & experimental systems

n/a	Involved in the study
☐	☒ Antibodies
☒	☐ Eukaryotic cell lines
☒	☐ Palaeontology
☐	☒ Animals and other organisms
☒	☐ Human research participants
☒	☐ Clinical data

Methods

n/a	Involved in the study
☒	☐ ChIP-seq
☒	☐ Flow cytometry
☒	☐ MRI-based neuroimaging

Antibodies

Antibodies used

PRIMARY ANTIBODIES
Chicken anti-GFP (Aves Labs, GFP-1020), rabbit anti-GFP (Invitrogen, A6455), rat anti-M2R (Synaptic Systems, 223017)
goat anti-nNOS (Abcam, ab1376), rabbit anti-NPY (Immunostar, 22940), goat anti-PV (Swant, pvg-214)
rat anti-SOM (Millipore, MAB354), goat anti-SOM (Santa Cruz, sc-7819), rabbit anti-GABA (Sigma, A2052).

SECONDARY ANTIBODIES (all from Jackson Immunoresearch)
Donkey anti-chicken Alexa 488 (703-545-155), donkey anti-rat Cy3 (712-165-150), donkey anti-sheep Dylight 647 (713-605-147), donkey anti-rabbit Dylight 594 (711-585-152), donkey anti-rat Alexa 594 (705-585-003).

Validation

PRIMARY ANTIBODIES
Sigma A2052: positive binding with GABA, and GABA-KLH in a dot blot assay, and negative binding with BSA.
Aves Labs GFP-1020: no staining observed in sections of GFP– brains from littermates.
Invitrogen A6455: no staining observed in sections of GFP– brains from littermates.
Synaptic Systems, 223017: no labelling in M2R knock-out mice.
Abcam ab1376: detects a band of ~160 kDa (predicted molecular weight: 161 kDa). Can be blocked with human nNOS peptide.
Immunostar 22940: staining is blocked by pre-absorption of the diluted antiserum with excess NPY. Absorption with other peptides does not reduce the intensity of staining.
Swant pvg-214: no labeling in PV knock-out mice
Millipore MAB354: no cross-reactivity to enkephalins, other endorphins, substance P or CGRP.
Santa Cruz sc-7819: band detected with Western blot in human Somatostatin-transfected 293 whole cell lysates but not in non-transfected ones.

SECONDARY ANTIBODIES
703-545-155: based on immunoelectrophoresis and/or ELISA, the antibody reacts with whole molecule chicken IgY. It also reacts with the light chains of other chicken immunoglobulins. No antibody was detected against non-immunoglobulin serum proteins.
712-165-150: based on immunoelectrophoresis and/or ELISA, the antibody reacts with whole molecule rat IgG. It also reacts with the light chains of other rat immunoglobulins. No antibody was detected against non-immunoglobulin serum proteins.
713-605-147: Based on immunoelectrophoresis and/or ELISA, the antibody reacts with whole molecule sheep IgG. It also reacts with the light chains of other sheep immunoglobulins. No antibody was detected against non-immunoglobulin serum proteins.
711-515-152: Based on immunoelectrophoresis and/or ELISA, the antibody reacts with whole molecule rabbit IgG. It also reacts with the light chains of other rabbit immunoglobulins. No antibody was detected against non-immunoglobulin serum proteins.
705-585-003: Based on immunoelectrophoresis and/or ELISA, the antibody reacts with whole molecule goat IgG. It also reacts with the light chains of other goat immunoglobulins. No antibody was detected against non-immunoglobulin serum proteins.

Specificity of secondary antibodies was further assessed by omitting the corresponding primary antibodies.

Animals and other organisms

Policy information about studies involving animals; ARRIVE guidelines recommended for reporting animal research

Laboratory animals

Mus musculus, transgenic lines:
- Dlx1/2CreER (Jackson Laboratory #014600)
- RCE:loxP (donated by Gord Fishell, Harvard Medical School)
- GAD67-GFP (donated by Hannah Monyer, University of Heidelberg)

All transgenic mouse lines were backcrossed to Swiss (SWR/J, Janvier labs) background for at least ten generations. Dlx1/2CreER +/-;RCE:LoxP+/+ male mice were crossed with wild-type Swiss females to generate Dlx1/2CreER+/-;RCE:LoxP+/- mice used for experiments.

Both male and female mice were used for experiments. For imaging experiments in neonatal mice, four to five days old (P4–P5) pups were used. For histological experiments in neonatal mice, P7 pups were used. For experiments in adult mice, 4-33 weeks old mice were used (mean age ± SD: P67 ± 32).

Wild animals

This study did not involve wild animals.

Field-collected samples

This study did not involve field-collected samples

October 2018

Ethics oversight	All protocols were performed under the guidelines of the French National Ethics Committee for Sciences and Health report on ''Ethical Principles for Animal Experimentation'' in agreement with the European Community Directive 86/609/EEC under agreement #01413.

Note that full information on the approval of the study protocol must also be provided in the manuscript.
