# Supplementary material for: Hippocampal hub neurons maintain distinct connectivity throughout their lifetime
Source: Nat Commun. 2020 Sep 11;11:4559. doi: 10.1038/s41467-020-18432-6 (PMC7486410; doi:10.1038/s41467-020-18432-6)
Supplement: Supplementary file 2 — Peer Review File [file 41467_2020_18432_MOESM2_ESM.docx]

Peer Review Information

**Journal:** Nature Communications

**Manuscript title:** Hippocampal hub neurons maintain distinct connectivity throughout their lifetime

**Corresponding author name(s):** Dr Rosa Cossart

**Editorial notes:**

**Transferred manuscripts**

This manuscript has been previously reviewed at another journal that is not operating a transparent peer review scheme. This document only contains reviewer comments and rebuttal letters for versions considered at *Nature Communications*.

**Reviewer comments & decisions:**

**Reviewer comments, first version:**

Reviewers' comments:

Reviewer #1 (Remarks to the Author):

The manuscript by Bocchio and colleagues explores the fate of early born GABAergic inhibitory interneurons in the hippocampal formation. This group has done pioneering research highlighting the important role of this early born population and the role they play in generating synchronous activity in the nascent hippocampus. One outstanding question has been what is the ultimate fate of this cell population on hippocampal maturation? Do they continue to play a role or is their existence terminate after their role in early stage development? In the present study the authors tackle this issue and provide very nice data that this diverse cell population persists into adulthood, adopts a morphology consistent with a long range projection profile and physiological experiments suggest that they receive differential innervation inside the CA1 hippocampus and are widely engage both during locomotor behavior and oscillatory activity. That these are difficult experiments performed on an ever elusive cell type should not be overlooked or undermined. As is typical of this lab, the experimental design and clarity of the results is first rate. That said while I am enthusiastic about the manuscript, many of the datasets are based on only a handful of recordings or experiments; simply because this is a small population and successful labeling and recording is a huge challenge. I think most of the conclusions are sound but the supporting data is often equivocal and although I am convinced by the authors conclusions I don’t think they nail home the actual cell type, its anatomy or neurochemical identity and the role they play in the adult circuit sufficiently enough. This somewhat weakens the story but my overall enthusiasm is high.

Figure 1 and related text. I am assuming that the cells shown in Figure 1b/d and f are different ebGABA cells? If so this should be pointed out in the legend to avoid confusion. Is the neurolucida drawing of the single cell the cell from which the sample data traces are taken? Again this should be stated for clarity.

Page 6. Line 73 “on the balance all ebGABAs could single-handedly……”. Do the authors really know that? What is the role for gap junction connectivity between hub cells etc. do the authors have evidence from this or previous studies that there is no evidence for a gap junction connected network at play here?

A better idea of the total number of ebGABAs typically labelled in a given animal would be helpful here. I imagine that the number is quite low but it’s difficult to gauge the numbers of cells that the authors are faced with in each slice at both early time points used for recordings as well as how many make it into adulthood. Is there a way that some type of quantification of ebGABA cell type can be provided between the two ages?

Related to this issue- the number of early born cells that function as hub cells appears to be extremely high and also to span multiple cell types based on immunohistochemical labeling. However the vast majority of these cells are born at much later time points as shown by others. The authors suggest in the discussion that COUPTFII may function as a transcription factor that could function as a temporal identity factor. Are ebGABAs in this study all positive for COUPTFII? Or any one marker for example (except GABA/GAD of course)? Do the authors really think that these cells destined to function as hub cells have numerous cell type identities that are consistent with later born interneuron but that they somehow segregate from them based on their birthdate?

Figure 2 and related text. Figure 2f shows a ctrlGABA interneuron. But what cell type comprises that group? It could be one of presumably dozens of interneuron types present in the CA1. Given that most interneuron subtypes share few common parameters is it useful to compare the properties of the ebGABA cells to an unknown population? I think if you were to simply show more features of the intrinsic properties of the ebGABA cells this would be more useful; who cares how they stack up against an unknown unhomogeneous control dataset ?

Figure 4. The use of the GAD mouse to probe the number of PV terminals onto the ebGABA population is reasonably convincing but the authros would be bewtter to determine whether the evoked GABA IPSC is sensitive to block by agatoxin or conotoxin which can discriminate between PV-inputs and CCK-inputs. This would provide a much cleaner conclusion as to whether the different IPSCs onto control versus eb cells truly reflects differences in PV input.

Figure 5 is perhaps the weakest part of the story and its really difficult to draw any major conclusions from this dataset despite the herculean task involved in executing this line of experiments. The bottom line that ebGABAs are highly involved in both locomotion and SCes is convincing but the number of sampled cells and the sparseness of the data make it hard to convince me that this is really a definitive observation.

Minor points:
For the uninitiated reader it would be helpful to explain the choice of the Dlx1/2-cre mouse line. Why was it chosen? Does it catch cells only from the MGE at this time point of tamoxifen administration etc…..

Figure 3 panels c/f & i. For clarity the authors should point out that the x axis represents somatic location of the recorded interneuron

Page 14 line 250 Lee et al 2014 should be replaced with a number


Reviewer #2 (Remarks to the Author):

This is a very interesting ms describing intrinsic, synaptic and microcircuit features of early born GABAergic cells from the CA1 region of the hippocampus. In general, results recapitulate similar data published by the lab regarding ebGABA hub cells from the CA3 region (Bonifazi et al., Science 2009). This new work complement this early report and further extend observations. The paper is a tour de force in trying to target the electrophysiological and morphological properties of this scarce population of cells in vitro and especially in vivo. I have some major comments and some additional suggestions to improve the ms.

Major comments

1. The narrative is not optimal, resulting in some interpretation problems. Given the goal of contrasting ebGABA cells (marked in Dlx1/2E7,5-GFP mice) with control GABA cells it is confusing the way data is presented. I would recommend authors to first provide estimations of the proportion ebGABA cells in the control population based on their distribution and features to better support interpretation of ctrlGABA cells and GDP data. Somehow, it reads like the order of Fig.2 and 1 (and associated sup.fig) should be interchanged. For instance, at the level of page 5, line 46-54, the reader may wonder what is the expected probability of having ebGABAergic cells in the ctrlGABAergic population. This is especially important given the low number of cells reported in this section. Similarly, in page 6, lines 68-77, it may be useful to know that info in order to more clearly interpret results from low sampling. I understand authors aimed to establish the hub role from the very beginning, but I feel data will be more robust if the distribution and properties of larger number of ebGABA cells are established before interpreting GDP data. In addition, in many parts of the ms, significant effect of ebGABA over ctrlGABA is established with low observation numbers; e.g. page 11 line 186 5/13 vs 1/14. Statistical significance should be reinforced.

2. ebGABA cells appear to be more typically driven by intra-hippocampal glutamatergic inputs than by input pathways. Authors made a good point on the radial organization of ebGABA cells, biased for deep layers, and indeed they also tested specific GABAergic inputs. However, nothing is reported regarding potential recruitment of ebGABA cells by local CA1 pyramidal cell axons. Given recent evidence of sublayer specific organization of CA1 microcircuits (Soltes’ and Prida’s lab) the question is relevant. Also, it may be interesting to know whether ebGABA cells in SO are targeted specifically by CA2 axons, which are also biased for deep CA1 layers (including deep pyramidal CA1 cells; Tonegawa lab).

3. Finally, while in vivo experiments are challenging and I very much appreciate the authors’ work, the low number of ebGABA cells (3 cells from 3 mice) recorded in vivo may be an issue. In addition, the type of information provided by these experiments is that ebGBA cells participate of assemble activity, and this is reported as a fraction of all cells in FOV. Given that only 1 cells is recorded per mice these numbers are more likely to reflect the effect of sampling (24 vs 77 vs 102) than a particular effect of ebGABA cells. Authors should try somehow to increase the yield and to twist analysis a bit more. For instance, is there any directionality in the interaction between ebGABA cells and the rest that could provide some evidence of hub functional architecture in vivo. Maybe using complementary opto-tagging experiments could help to provide additional cues.

Other comments

4. All in vitro recordings are from intermediate/ventral hippo (horizontal slices) but in vivo imaging targets the dorsal hippocampus. The ms will benefit from targeting some of the dorsal ebGABA cells in coronal slices (if GDPs cannot be evaluated in these sections, some basic intrinsic and synaptic properties can be added to better support transitions to in vivo imaging)
5. The distribution and location, and some properties of ebGABA cells are reminiscent of some nNOS-expressing long-range GABAergic cells recently identified with intersectional approaches (Wick et al., eLife 2019). This nNOS population is born later than the ebGABA cells included in the study, but it may be useful to comment on their potential similarities and differences.
6. Axonal arbors of the ebGABA cells in Fig.2 appear similar to the crtlGABA cell in Fig.1. There are better morphologies in Supp.Fig.1.
7. Please, show single dot data in Fig.2a,b to better appreciate estimation statistics
8. Ref 11 and 15 are the same
9. 40% of ebGABA cells are long-range projections. What about the other 60% of them?
10. Please check grammar and wording (single-handedly?)


Reviewer #3 (Remarks to the Author):

In this paper, Bocchio et al. extend previous work from their lab on GABAergic hub cells in the hippocampus. The authors show now new data arguing the following three points: (1) early-born GABAergic (ebGABA) neurons in the hippocampal area CA1 exhibit, like ebGABA neurons in CA3, hub-cell like features during development; (2) even later in the adult brain, these ebGABA cells remain distinct in terms of their (i) molecular and morphological features, (ii) connectivity and, (iii) intrinsic firing properties; and (3) the activity of these ebGABA neurons is correlated with the activity of neuronal ensembles in CA1 in vivo (e.g., during sharp wave ripples) and are involved in organizing the population activity CA1. I have several concerns/questions that need to be addressed:

1) Throughout the paper, the number of neurons that show any effect when stimulated and/or are included at all in the study is very low. Only n=6 CA1 early-born neurons (out of 65) were found to affect giant depolarizing potentials (GDPs) in vitro, only n=3 neurons were found and recorded from in 39 (!) animals in vivo. These numbers are considerably lower compared to the numbers reported previously for CA3 by the same group (Bonifazi et al., 2009, Science; Picardo et al., 2011, Neuron). Along the same lines, the authors do not show any evidence that these ebGABA cells operate in fact as hub cells in CA1 (see experiments in Bonifazi et al., figure 4c), and figure 1b seems to show the opposite (although this is, of course, just a single example). Taken together, these results suggest that, perhaps, there is a fundamental difference in hub cell prevalence and function between CA1 and CA3. CA3 is recurrently connected and known to be a major initiation site for GDPs. But what is the significance of having hub cells in CA1?

2) It is not very transparent how the authors decided to include or exclude individual neurons in the various analyses (e.g., 18 ebGABA neurons were included in the axon length analysis in figure 1i , but only 6 were included in figure 1g). Is there an overlap between these populations? The authors should provide a clear rationale for including/excluding data.

3) Figure 1c&d shows already for the pre-stimulation condition a difference of about 10s in the inter-GDP interval between the ctrl and the ebGABA stimulation case. This difference is comparable to the difference between pre-stimulation and during ebGABA neuron stimulation. Were these experiments performed in the same or different mouse genotypes? The authors should show that GDPs features and frequencies do not differ between mouse genotypes. Also, a quantification across all experiments should be included.

4) How exactly are the colorplots in figure 1e&f computed?

5) Overall, the morphological reconstructions show a considerable diversity within the ebGABA group (see figure 1h, 2e, Suppl. Fig. 5). Is there any systematic difference in terms of morphology, molecular identify, or firing properties between those ebGABA neurons that affected GDPs and those that did not? Also, the reconstructions seem overall incomplete, and, maybe as a result, the spread of measured values is wide (Figure 1i). It is indeed hard to accurately determine the dendritic and axonal lengths of interneurons in slices as their processes are regularly cut off during slicing. Is it possible to label these ebGABA neurons in vivo (e.g., GFP instead of biocytin) so that more complete reconstructions can be obtained?

6) Figure 5 shows a correlation between the activity of the ebGABA neurons and of neuronal ensembles in CA1 in adult mice. Yet, the authors write in the paper that these CA1 hub cells seem to have very little axonal arborization within CA1 (e.g., p.9, line 137-139) and tend to project to extra-hippocampal targets. The authors should provide evidence or at least discuss how these ebGABA neurons (especially given their low prevalence and their specific projection patterns) could affect CA1 activity in vivo at all.

**Author rebuttal, first version:**

***Reviewer #1 (Remarks to the Author)***

*The manuscript by Bocchio and colleagues explores the fate of early born GABAergic inhibitory interneurons in the hippocampal formation. This group has done pioneering research highlighting the important role of this early born population and the role they play in generating synchronous activity in the nascent hippocampus. One outstanding question has been what is the ultimate fate of this cell population on hippocampal maturation? Do they continue to play a role or is their existence terminate after their role in early stage development? In the present study the authors tackle this issue and provide very nice data that this diverse cell population persists into adulthood, adopts a morphology consistent with a long range projection profile and physiological experiments suggest that they receive differential innervation inside the CA1 hippocampus and are widely engage both during locomotor behavior and oscillatory activity. That these are difficult experiments performed on an ever elusive cell type should not be overlooked or undermined. As is typical of this lab, the experimental design and clarity of the results is first rate. That said while I am enthusiastic about the manuscript, many of the datasets are based on only a handful of recordings or experiments; simply because this is a small population and successful labeling and recording is a huge challenge. I think most of the conclusions are sound but the supporting data is often equivocal and although I am convinced by the authors conclusions I don’t think they nail home the actual cell type, its anatomy or neurochemical identity and the role they play in the adult circuit sufficiently enough. This somewhat weakens the story but my overall enthusiasm is high.*

We thank the reviewer for the positive feedback on our manuscript. We have performed new experiments and analyses to address his/her concerns. We have added another figure (Figure 1) that better illustrates the distribution of early-born GABAergic neurons in the developing and adult CA1. We have also clarified various parts of the text and figure legends. We hope that this revised version of the paper provides a stronger support to our conclusions about the properties of adult early-born GABA neurons and the role they play in the mature hippocampus.

1) *Figure 1 and related text. I am assuming that the cells shown in Figure 1b/d and f are different ebGABA cells? If so this should be pointed out in the legend to avoid confusion. Is the neurolucida drawing of the single cell the cell from which the sample data traces are taken? Again this should be stated for clarity.*

Panels b/d represent the same cells. All the examples in other panels are from different
cells. We have added this information to the figure legend. This figure is now Figure 2.

0) *Page 6. Line 73 “on the balance all ebGABAs could single-handedly......”. Do the authors really know that? What is the role for gap junction connectivity between hub cells etc. do the authors have evidence from this or previous studies that there is no evidence for a gap junction connected network at play here?*

We have not tested whether ebGABAs form gap junctions with other GABAergic cells. We assumed that gap junctions between ebGABAs were unlikely given the scarcity of these cells in CA1. However, we cannot fully exclude this possibility because many ebGABAs have extensive dendritic trees. Additionally, it is possible that SOM+ ebGABAs form gap junctions with other SOM+ cells. Thus, we have removed the word “single-handedly” from the sentence (now at page 10, line 152-155.)

3) *A better idea of the total number of ebGABAs typically labelled in a given animal would be helpful here. I imagine that the number is quite low but it’s difficult to gauge the numbers of cells that the authors are faced with in each slice at both early time points used for recordings as well as how many make it into adulthood. Is there a way that some type of quantification of ebGABA cell type can be provided between the two ages?*

We have illustrated and quantified the amount of ebGABAs found per section in early postnatal development and adulthood. We have performed quantifications in both fixed 70-80 μm sections (Figure 1 and pages 4-6) and in acute slice experiments performed at neonatal stage (page 7, lines 84-90) and adult stage (page 15, lines 248-250).

4) *Related to this issue- the number of early born cells that function as hub cells appears to be extremely high and also to span multiple cell types based on immunohistochemical labeling. However the vast majority of these cells are born at much later time points as shown by others. The authors suggest in the discussion that COUPTFII may function as a transcription factor that could function as a temporal identity factor. Are ebGABAs in this study all positive for COUPTFII? Or any one marker for example (except GABA/GAD of course)? Do the authors really think that these cells destined to function as hub cells have numerous cell type identities that are consistent with later born interneuron but that they somehow segregate from them based on their birthdate?*

We have tested the expression of COUP-TFII in ebGABAs (page 14, lines 224-227 and Supplementary Figure 3h-i). However, only 1/26 ebGABAs was found immunopositive, suggesting that this transcription factor isn’t a common marker of this population. We have re-written the discussion accordingly (page 33 line 615-618).

As the reviewer points out, ebGABAs are neurochemically diverse. Thus, present evidence suggests that they are not part of one cell type. However, we do not think that most ebGABAs have “cell type identities that are consistent with later born interneurons”. While they do share some broad markers with other cell types (e.g. SOM or PV), most of their “cell type identities” remain elusive. For instance, ~30% of ebGABAs around the pyramidal layer express PV, but we haven’t found evidence of basket-like or chandelier-like axonal arbors. In addition, many ebGABAs in superficial CA1 layers were immunonegative for most molecular markers tested. Thus, our current hypothesis is that ebGABAs are formed by multiple subtypes (expressing different markers and potentially projecting to different sites). Most of these subtypes share commonalities (e.g. long-range projections, little PV+ inputs) and do not overlap with known interneuron types.

5) *Figure 2 and related text. Figure 2f shows a ctrlGABA interneuron. But what cell type comprises that group? It could be one of presumably dozens of interneuron types present in the CA1. Given that most interneuron subtypes share few common parameters is it useful to compare the properties of the ebGABA cells to an unknown population? I think if you were to simply show more features of the intrinsic properties of the ebGABA cells this would be more useful; who cares how they stack up against an unknown unhomogeneous control dataset ?*

We agree that a downside of using random GABAergic cells as a control group is the unknown identity of control cells and their high variability. We have decided to compare ebGABAs to random GABAergic cells because differences between the two groups are likely to reveal parameters based on which ebGABAs strongly segregate from most inhibitory cells. This is relevant because the vast majority of ctrlGABAs are local interneurons, while many ebGABAs may be projection cells. Another option was to compare ebGABAs with another extreme (e.g. late-born cells) or with a homogeneous class (e.g. PV+ basket cells). This might have yielded more differences, but we reckon that it would have been less informative on the uniqueness of this cell population. Given that we have used random interneurons as control group for many experiments, we prefer to keep this comparison even for intrinsic electrophysiological parameters. Nonetheless, we have described the main intrinsic features of ebGABAs in the text (page 15, lines 251-254)

**Rebuttal Figure 1. (a)** Innervation of a representative ebGABA by CB1+ terminals.

(a1) maximum projection of confocal stack.
(a2) single optical section highlighting single boutons (arrows). **(b)** Innervation of ctrlGABAs by CB1+ terminals (maximum projection of confocal stack). *Inset*: single optical section showing two ctrlGABAs in stratum pyramidale innervated by single CB1+ boutons (arrows).

*6)	Figure 4. The use of the GAD mouse to probe the number of PV terminals onto the*

*ebGABA population is reasonably convincing but the authors would be better to determine whether the evoked GABA IPSC is sensitive to block by agatoxin or conotoxin which can discriminate between PV-inputs and CCK-inputs. This would provide a much cleaner conclusion as to whether the different IPSCs onto control versus eb cells truly reflects differences in PV input.*

To verify whether ebGABAs receive distinct innervation from other interneuron types compared to ctrlGABAs, we have performed CB1 immunostainings in Dlx1/2(E7.5)-GFP mice and GAD67-GFP mice to quantify the number of boutons received by both classes from CCK+ interneurons (see Rebuttal Figure 1 below).

Unfortunately, the ongoing pandemic and lockdown measures in place in France have prevented us from performing a detailed quantification like in the case of PV+ inputs. While both ctrlGABAs and ebGABAs appear to receive CB1+ inputs, we could not assess whether the number of CB1+ terminals differ between the two populations. Thus, we have added in the discussion that “since superficial pyramidal cells receive little PV innervation but abundant inputs from CCK+ interneurons, future work could establish whether ebGABAs receive preferential innervation from CCK+ interneurons.” (page 31, lines 572­574).

7) *Figure 5 is perhaps the weakest part of the story and its really difficult to draw any major conclusions from this dataset despite the herculean task involved in executing this line of experiments. The bottom line that ebGABAs are highly involved in both locomotion and SCes is convincing but the number of sampled cells and the sparseness of the data make it hard to convince me that this is really a definitive observation.*

We acknowledge this was the weakest, but most challenging part of the story and therefore decided to focus on additional *in vivo* experiments (three months were necessary to increase significantly our dataset) and analyses. We have imaged additional ebGABAs, reaching a total of 9 cells (7 imaged from the stratum pyramidale and 2 from the stratum oriens, from 7 FOVs from 6 mice). This confirmed that the majority of ebGABAs are activated with locomotion (9/9 cells), SCEs (8/9 cells), assembly activity (8/9 cells) and all three events combined (7/9 cells). As a population, ebGABAs were significantly more likely to be activated by these phenomena than random control cells (with the exception of SCEs, for which most control cells also showed activation). Interestingly, we have also performed functional connectivity analysis and found that ebGABAs exhibit stronger functional output connectivity than random control cells. These data are illustrated in Figure 6 and described on pages 24-28. Overall, these data demonstrate that ebGABAs maintain a strong link with network activity even in adulthood *in vivo*.

*Minor points:*

8) *For the uninitiated reader it would be helpful to explain the choice of the Dlx1/2-cre mouse line. Why was it chosen? Does it catch cells only from the MGE at this time point of tamoxifen administration etc*

We have followed the reviewer’s suggestion and described the rationale for using this transgenic line at page 4, lines 36-45.

9) *Figure 3 panels c/f & i. For clarity the authors should point out that the x axis represents somatic location of the recorded interneuron*

We have added this information in the figure legend (please note that old Figure 3 is now Figure 4).

10) *Page 14 line 250 Lee et al 2014 should be replaced with a number* Corrected.

***Reviewer #2 (Remarks to the Author):***

*This is a very interesting ms describing intrinsic, synaptic and microcircuit features of early born GABAergic cells from the CA1 region of the hippocampus. In general, results recapitulate similar data published by the lab regarding ebGABA hub cells from the CA3 region (Bonifazi et al., Science 2009). This new work complement this early report and*

*further extend observations. The paper is a tour de force in trying to target the electrophysiological and morphological properties of this scarce population of cells in vitro and especially in vivo. I have some major comments and some additional suggestions to improve the ms.*

We are glad that the reviewer appreciated the significance of our manuscript. We thank him/her for the positive feedback.

*Major comments*

1) *The narrative is not optimal, resulting in some interpretation problems. Given the goal of contrasting ebGABA cells (marked in Dlx1/2E7,5-GFP mice) with control GABA cells it is confusing the way data is presented. I would recommend authors to first provide estimations of the proportion ebGABA cells in the control population based on their distribution and features to better support interpretation of ctrlGABA cells and GDP data. Somehow, it reads like the order of Fig.2 and 1 (and associated sup.fig) should be interchanged. For instance, at the level of page 5, line 46-54, the reader may wonder what is the expected probability of having ebGABAergic cells in the ctrlGABAergic population. This is especially important given the low number of cells reported in this section. Similarly, in page 6, lines 68-77, it may be useful to know that info in order to more clearly interpret results from low sampling. I understand authors aimed to establish the hub role from the very beginning,*

*but I feel data will be more robust if the distribution and properties of larger number of ebGABA cells are established before interpreting GDP data. In addition, in many parts of the ms, significant effect of ebGABA over ctrlGABA is established with low observation numbers; e.g. page 11 line 186 5/13 vs 1/14. Statistical significance should be reinforced.*

We have followed the reviewer’s suggestion. We have added an additional figure (Figure 1, described in the results at pages 4-7) illustrating the estimated proportion of ebGABAs in the GABA+ cell population (~1%). We have also highlighted the difference in the amount of hippocampal ebGABAs and the amount of SOM+ cells (the main

neurochemical marker expressed by these neurons). EbGABAs are 20 times less abundant than all SOM+ cells. The figure also shows that the numbers of CA1 ebGABAs are similarly small both at neonatal stage and in adulthood.

We have imaged additional ebGABAs *in vivo*, reaching a total of 9 cells and confirming previous results (see point 3 below for details). For the experiments in which we

stimulated the ventromedial thalamus or the entorhinal cortex, we recognize that numbers may be low to draw unambiguous conclusions for each pathway. However, we did not compare the two cell populations for single pathways, but only for intra-hippocampal (CA3) and extra-hippocampal (thalamus and entorhinal cortex pooled) inputs. Besides the difference in the proportion of responsive cells (Figure 4j), we now show an additional difference in maximum EPSC amplitude for extra-hippocampal afferents (Figure 4l).

6) *ebGABA cells appear to be more typically driven by intra-hippocampal glutamatergic inputs than by input pathways. Authors made a good point on the radial organization of ebGABA cells, biased for deep layers, and indeed they also tested specific GABAergic inputs. However, nothing is reported regarding potential recruitment of ebGABA cells by local CA1 pyramidal cell axons. Given recent evidence of sublayer specific organization of CA1 microcircuits (Soltes’ and Prida’s lab) the question is relevant. Also, it may be interesting to know whether ebGABA cells in SO are targeted specifically by CA2 axons, which are also biased for deep CA1 layers (including deep pyramidal CA1 cells; Tonegawa lab).*

We agree with the reviewer: testing how CA1 and CA2 inputs contribute to the excitation of ebGABAs is a very important experiment. We had planned these experiments, but gave priority to the *in vivo* imaging experiments because more technically challenging. Unfortunately, the ongoing pandemic and lockdown measures in place in France prevent us from performing these experiments in the nearby future. Thus, we have added in the discussion that “future experiments should probe the contribution of CA1 and CA2 pyramidal cells to the excitation of ebGABAs” (page 30, lines 561-564).

3) *Finally, while in vivo experiments are challenging and I very much appreciate the authors’ work, the low number of ebGABA cells (3 cells from 3 mice) recorded in vivo may be an issue. In addition, the type of information provided by these experiments is that ebGBA cells participate of assemble activity, and this is reported as a fraction of all cells in FOV. Given that only 1 cells is recorded per mice these numbers are more likely to reflect the effect of sampling (24 vs 77 vs 102) than a particular effect of ebGABA cells. Authors should try somehow to increase the yield and to twist analysis a bit more. For instance, is there any directionality in the interaction between ebGABA cells and the rest that could provide some evidence of hub functional architecture in vivo. Maybe using complementary opto-tagging experiments could help to provide additional cues.*

We have performed additional *in vivo* experiments and analyses. We have imaged additional ebGABAs, reaching a total of 9 cells (7 imaged from the stratum pyramidale and 2 from the stratum oriens, from 7 FOVs from 6 mice). This confirmed that the majority of ebGABAs are activated with locomotion (9/9 cells), SCEs (8/9 cells), assembly activity (8/9 cells) and all three events combined (7/9 cells). As a population, ebGABAs were significantly more likely to be activated by these phenomena than random control cells (with the exception of SCEs, for which most control cells also showed activation). We have also performed functional connectivity analysis and found that ebGABAs exhibit stronger functional output connectivity than random control cells. These data are illustrated in Figure 6 and described at page 28, line 502-513.

*Other comments*

4) *All in vitro recordings are from intermediate/ventral hippo (horizontal slices) but in vivo imaging targets the dorsal hippocampus. The ms will benefit from targeting some of the dorsal ebGABA cells in coronal slices (if GDPs cannot be evaluated in these sections, some basic intrinsic and synaptic properties can be added to better support transitions to in vivo imaging)*

All slice recordings performed in adulthood involved sampling from both the dorsal and intermediate/ventral hippocampus in coronal slices. We apologize if this was not sufficiently clear. We have written this explicitly in the Results (page 15, lines 247-248) and in the Methods sections (page 40, lines 775-776).

5) *The distribution and location, and some properties of ebGABA cells are reminiscent of some nNOS-expressing long-range GABAergic cells recently identified with intersectional approaches (Wick et al., eLife 2019). This nNOS population is born later than the ebGABA cells included in the study, but it may be useful to comment on their potential similarities and differences.*

We have discussed this at page 32, lines 589-592.

6) *Axonal arbors of the ebGABA cells in Fig.2 appear similar to the crtlGABA cell in Fig.1. There are better morphologies in Supp.Fig.1.*

Please note that reconstructions in Supplementary Figure 1 are from ebGABAs recorded at neonatal stage. Reconstructed cells that were recorded in adulthood are illustrated in Supplementary Figure 4. We would prefer to keep the cells illustrated in old Figure 2 (now Figure 3) because they seem to represent well the poor local arborization of ebGABAs at adult stages.

7) *Please, show single dot data in Fig.2a,b to better appreciate estimation statistics*

These panels now show single data points. Please note that panel a has been moved to Figure 1e, whereas panel b is now in Figure 3a.

0) *Ref 11 and 15 are the same*

We thank the reviewer for spotting this mistake. We have corrected it.

*9)	40% of ebGABA cells are long-range projections. What about the other 60% of them?*

We have estimated this percentage by matching ebGABAs to known GABAergic populations based on neurochemical markers. This may be a rather conservative estimation because none of the biocytin-filled ebGABAs had dense local axonal arbors and could be assigned to a known class of interneurons. Additionally, hippocampal long-range-projecting PV+ cells have been reported (Lübkemann *et al.*, 2015; Christenson Wick *et al.*, 2017), thus PV+ ebGABAs in the stratum pyramidale may also be projection cells (we found no evidence of basket, chandelier or bistratified cells). However, we prefer to provide a conservative percentage for two reasons. First, the neurochemical profile of many ebGABAs (particularly the ones located in stratum radiatum and lacunosum-moleculare) remains unclear. Second, the reconstructions of the cells filled *in vitro* are likely incomplete. *In vivo* tracing is necessary to make more confident estimations.

10) *Please check grammar and wording (single-handedly?)*

We have removed this word, also because of a conceptual issue regarding possible gap junctions (see point 2 of Reviewer 1).

***Reviewer #3 (Remarks to the Author):***

*In this paper, Bocchio et al. extend previous work from their lab on GABAergic hub cells in the hippocampus. The authors show now new data arguing the following three points: (1) early-born GABAergic (ebGABA) neurons in the hippocampal area CA1 exhibit, like ebGABA neurons in CA3, hub-cell like features during development; (2) even later in the adult brain, these ebGABA cells remain distinct in terms of their (i) molecular and morphological features, (ii) connectivity and, (iii) intrinsic firing properties; and (3) the activity of these ebGABA neurons is correlated with the activity of neuronal ensembles in CA1 in vivo (e.g., during sharp wave ripples) and are involved in organizing the population activity CA1. I have several concerns/questions that need to be addressed:*

We thank the reviewer for the constructive criticism that led us to improve our manuscript with additional experiments, analyses and clarifications.

*1)	Throughout the paper, the number of neurons that show any effect when stimulated and/or are included at all in the study is very low. Only n=6 CA1 early-born neurons (out of 65) were found to affect giant depolarizing potentials (GDPs) in vitro, only n=3 neurons were found and recorded from in 39 (!) animals in vivo. These numbers are considerably lower*

*compared to the numbers reported previously for CA3 by the same group (Bonifazi et al., 2009, Science; Picardo et al., 2011, Neuron). Along the same lines, the authors do not show any evidence that these ebGABA cells operate in fact as hub cells in CA1 (see experiments in Bonifazi et al., figure 4c), and figure 1b seems to show the opposite (although this is, of course, just a single example). Taken together, these results suggest that, perhaps, there is a fundamental difference in hub cell prevalence and function between CA1 and CA3. CA3 is recurrently connected and known to be a major initiation site for GDPs. But what is the significance of having hub cells in CA1?*

We believe that these issues mainly arise from lack of clarity in the manuscript. We have improved the description of the experiments and the findings to avoid confusions. Regarding the GDP experiments, we could test the effect of ebGABA stimulation on the network only in a minority of cases (6 cells from 6 mice) because in many experiments we could not detect spontaneous GDPs. This issue was probably due to the fact that at the beginning of the project we tended to sacrifice Cre-negative pups, resulting in smaller litters and faster growth rate of Cre-positive pups. This led to higher weight at P4-P5 and fewer GDPs. Keeping the entire litter seemed to solve this issue. We have nonetheless reported the entire number of cells and slices used to complete this dataset. Thus, 6/6 ebGABAs (and not 6/65) had an effect on GDPs.

Performing functional connectivity analysis in *in vitro* imaging experiments was prevented (as discussed in the Results) by the fact that – for unknown reasons – ebGABAs could not be filled with the calcium indicator Fura2-AM. This was the case also for CA3 ebGABAs in our previous paper (Picardo *et al.*, 2011).

For *in vivo* experiments, we previously reported the total number of mice needed to achieve 3 imaged ebGABAs. We have now included only the mice that displayed a clear field-of-view and visible jRGECO1a expression in CA1 cells. We have also performed additional experiments. In total, we imaged 17 ebGABAs from 15 mice. However, we discarded 8 cells because excessive movement in the z-axis (4 cells), no expression of jRGECO1a in the Dlx1/2(E7.5)-GFP+ cells (3 cells) or epileptic-like activity detected in the FOV (1 cell) prevented a meaningful analysis. Thus, the current manuscript includes analysis from 9 ebGABAs imaged *in vivo*. All these details are present at page 25, lines 442-449.

2) *It is not very transparent how the authors decided to include or exclude individual neurons in the various analyses (e.g., 18 ebGABA neurons were included in the axon length analysis in figure 1i , but only 6 were included in figure 1g). Is there an overlap between these populations? The authors should provide a clear rationale for including/excluding data.*

Following up to our previous reply to point 1, we discarded ebGABAs from the GDP analysis if no GDPs were detected in baseline conditions. However, we kept these cells for anatomical analyses. Reconstructions and morphometric analyses were performed in a subset of ebGABAs (*n* = 18), which include all 6 cells for which GDP experiments could be analyzed.

3) *Figure 1c&d shows already for the pre-stimulation condition a difference of about 10s in the inter-GDP interval between the ctrl and the ebGABA stimulation case. This difference is comparable to the difference between pre-stimulation and during ebGABA neuron stimulation. Were these experiments performed in the same or different mouse genotypes? The authors should show that GDPs features and frequencies do not differ between mouse genotypes. Also, a quantification across all experiments should be included.*

We thank the reviewer for raising this issue. Ensuring that no baseline difference is present between the two cell populations is indeed an important control. CtrlGABA experiments were performed in littermate Cre-negative pups. The difference between ebGABAs and ctrlGABAs is unlikely to arise from spontaneous differences in GDP rate between Cre-positive and Cre-negative mice because the median GDP intervals of ctrlGABA and ebGABA experiments were comparable (ctrlGABA: median 0.02 Hz, IQR 0.014 - 0.022 Hz; ebGABA: median 0.017 Hz, IQR 0.015 – 0.023 Hz, P > 0.99, Mann-Whitney U test). We have added this to the results (page 8, lines 103-107)

4) *How exactly are the colorplots in figure 1e&f computed?*

Colorplots in old Figure 1e-f (current Figure 2e-f) are raster plots of calcium transient onsets following each stimulation (each row represents the average activity of a cell across 20 trials). Colorbars depict the number of onsets. We have further clarified the figure legend.

5) *Overall, the morphological reconstructions show a considerable diversity within the ebGABA group (see figure 1h, 2e, Suppl. Fig. 5). Is there any systematic difference in terms of morphology, molecular identify, or firing properties between those ebGABA neurons that affected GDPs and those that did not? Also, the reconstructions seem overall incomplete, and, maybe as a result, the spread of measured values is wide (Figure 1i). It is indeed hard to accurately determine the dendritic and axonal lengths of interneurons in slices as their processes are regularly cut off during slicing. Is it possible to label these ebGABA neurons in vivo (e.g., GFP instead of biocytin) so that more complete reconstructions can be obtained?*

All ebGABAs for which effect of stimulation on GDPs could be analyzed (6/6) had an effect on either GDP frequency, GDP onset or both. Interestingly, we found that cells exerting a hub function in the network (affecting either GDP frequency or GDP synchronization, *n* = 7, formed by 1 ctrlGABA and 6 ebGABAs) display significantly longer axons but not dendrites than non-hub cells (7 ctrlGABAs, please see

Supplementary Figure 2c-d). This suggests that a widespread axonal arborization is linked to the ability of a GABAergic neuron to control GDP generation.

We agree with the reviewer that reconstructions from cells that have been filled in slices are likely incomplete. We have discussed this at page 32, lines 593-601 and page 33, lines 625-626. We have attempted to trace the axons of single Dlx1/2(E7.5)-GFP+ cells in clarified brains. While we have been able to trace axons successfully using a strong fluorophore (tdTomato), the GFP signal of the Dlx1/2(E7.5)-GFP line is too weak and we were not able to trace the axons of ebGABAs. We prefer to avoid using an *Ai14* reporter line to reveal ebGABAs with tdTomato because this reporter line led to significant unspecific labelling in our hands (see Villette *et al.*, 2016).

*6	Figure 5 shows a correlation between the activity of the ebGABA neurons and of neuronal ensembles in CA1 in adult mice. Yet, the authors write in the paper that these CA1 hub cells seem to have very little axonal arborization within CA1 (e.g., p.9, line 137-139) and tend to project to extra-hippocampal targets. The authors should provide evidence or at least discuss how these ebGABA neurons (especially given their low prevalence and their specific projection patterns) could affect CA1 activity in vivo at all.*

This is a very important point. The correlation between ebGABA activity and CA1 assemblies may not necessarily reflect modulation of CA1 activity by ebGABAs. It could also be that ebGABAs are excited (and fired) by co-active CA1 pyramidal cells (or by a common input such as CA3). However, we have added a new analysis showing that

ebGABAs display strong functional connectivity *in vivo*. This is illustrated in Figure 6j and Supplementary Figure 8, and described at page 28, lines 502-513. Indeed, this may seem at odds with the finding that ebGABAs’ axons arborize little in CA1. EbGABA’s axons could still innervate several CA1 cells but through arborization in different planes that are largely severed in acute coronal slices. Alternatively, local projections could be minimal but circuit *motifs* could guarantee a powerful effect on the CA1 network. These could be local disinhibition (i.e. inhibition of few CA1 interneurons targeting many pyramidal cells, such as basket cells) or inhibition of other regions that project to CA1 (such as the medial septum or CA3). Future experiments should reveal the axonal fields of ebGABAs labelled in vivo, as well as their postsynaptic targets. We have added these considerations to the discussion (at page 32, lines 593-601 and page 33, lines 625-626.).

**References**

Christenson Wick, Z., Leintz, C.H., Xamonthiene, C., Huang, B.H., & Krook-Magnuson, E. (2017) Axonal sprouting in commissurally projecting parvalbumin-expressing interneurons. *J. Neurosci. Res.*, **95**, 2336–2344.

Lübkemann, R., Eberhardt, J., Röhl, F.-W., Janitzky, K., Nullmeier, S., Stork, O., Schwegler, H., & Linke, R. (2015) Identification and Characterization of GABAergic Projection Neurons from Ventral Hippocampus to Amygdala. *Brain Sci.*, **5**, 299–317.

Picardo, M.A., Guigue, P., Bonifazi, P., Batista-Brito, R., Allene, C., Ribas, A., Fishell, G., Baude, A., & Cossart, R. (2011) Pioneer GABA Cells Comprise a Subpopulation of Hub Neurons in the Developing Hippocampus. *Neuron*, **71**, 695–709.

Villette, V., Guigue, P., Picardo, M.A., Sousa, V.H., Leprince, E., Lachamp, P., Malvache, A., Tressard, T., Cossart, R., & Baude, A. (2016) Development of early-born γ-Aminobutyric acid hub neurons in mouse hippocampus from embryogenesis to adulthood. *J. Comp. Neurol.*, **524**, 2440–2461.

**Reviewer comments, second version:**

Reviewer #1 (Remarks to the Author):

No further comments


Reviewer #2 (Remarks to the Author):

Authors have done a serious revision with new experiments and analyses. I found the inclusion of Fig.1 very helpful in establishing the expected proportion and distribution of ebGABAergic CA1 cells and in informing next experiments. Neurochemical characterization of ebGABAergic cells is very complelling. Similarly, in vivo experiments are reinforced with additional numbers and analyses.

Overall, the ms has improved significantly.

Some remaining details are:

1- page 5 line 47; please specify how many sections per brain, otherwise it reads as if the entire structure was counted, which I guess is not the case.

2- same page, line 56: same comment here; why 6 brains versus 4 brains in the previous paragraph? bilateral vs unilateral counting? Please, clarify

3- Fig.3: the sublinear I/O relatioship of ebGABAergic cells seems to be very dependent on a depolarizing block, which authors mention in the text. This could be an artifactual response to large current injections (>600 nA) and less likely to takes place physiologically. However, the rastergram and I/O curve suggest it also occurs for intermediate current pulses (e.g. 500 nA). Could authors add the trace for such intermediate pulses for comparison?

4- It is unfortunate that the COVID situation prevented authors to test for CA2 inputs. The preferential location of ebGABAergic CA1 cells at SO makes the possibility of a major innervation by CA2 very likely. I would not like to stress authors with hard demands at this point, but I feel this issue deserves more serious consideration. At least the note in Discussion could be more clear (maybe it can be written in terms of prediction?)

5- Network graph analysis of in vivo data is very useful (Fig.6j) but only 2/7 cells exhibited hub-like dynamics. Possibly, evaluating this locally will be always elusive for a long-range population of cells. Is the connectivity analysis implemented in the whole recording window independently on the state? What if the analysis is focused in periods of locomotion or around the start of locomotion? Would that increase the chances to capture hub-like dynamics in more cells? Anyway, given the small proportion (2/7) I would recommend authors to implement some form of surrogate analysis to discard chance results in fig.6i.

6- Fig.6k, It is unclear what the different shapes of the violin like plots represent. Please help the reader to understand the plot.


Reviewer #3 (Remarks to the Author):

The authors have done a great job of addressing my comments, in particular, when taking into account the coronavirus crisis. In particular, the in vivo recordings are extremely difficult and heroic, and the conclusion that the ebGABA neurons are highly active during locomotion, SCEs, and assembly activity is convincing. The evidence that these neurons are hub cells appears sounds as well, although the number of neurons is still low.

**Author rebuttal, second version:**

**Reviewer #2 (Remarks to the Author):**

*Authors have done a serious revision with new experiments and analyses. I found the inclusion of Fig.1 very helpful in establishing the expected proportion and distribution of ebGABAergic CA1 cells and in informing next experiments. Neurochemical characterization of ebGABAergic cells is very complelling. Similarly, in vivo experiments are reinforced with additional numbers and analyses.*

*Overall, the ms has improved significantly.
Some remaining details are:*

1- *page 5 line 47; please specify how many sections per brain, otherwise it reads as if the entire structure was counted, which I guess is not the case.*

We have added this information.

2- *same page, line 56: same comment here; why 6 brains versus 4 brains in the previous paragraph? bilateral vs unilateral counting? Please, clarify*

We have added the number of sections. Six brains from adult mice were used to quantify the distribution of ebGABAs (coronal sections). The four brains mentioned in the paragraph above (P7 mice) were used to quantify the density of ebGABAs in the entire hippocampus in comparison to GABA+ and SOM+ cells.

3- *Fig.3: the sublinear I/O relatioship of ebGABAergic cells seems to be very dependent on a depolarizing block, which authors mention in the text. This could be an artifactual response to large current injections (>600 nA) and less likely to takes place physiologically. However, the rastergram and I/O curve suggest it also occurs for intermediate current pulses (e.g. 500 nA). Could authors add the trace for such intermediate pulses for comparison?*

We have added the trace with intermediate current amplitude as the Reviewer suggested.

4- *It is unfortunate that the COVID situation prevented authors to test for CA2 inputs. The preferential location of ebGABAergic CA1 cells at SO makes the possibility of a major innervation by CA2 very likely. I would not like to stress authors with hard demands at this point, but I feel this issue deserves more serious consideration. At least the note in Discussion could be more clear (maybe it can be written in terms of prediction?)*

We have added some hypotheses on the involvement of CA2 at page 31, line 570.

5- *Network graph analysis of in vivo data is very useful (Fig.6j) but only 2/7 cells exhibited hub-like dynamics. Possibly, evaluating this locally will be always elusive for a long-range population of cells. Is the connectivity analysis implemented in the whole recording window independently on the state? What if the analysis is focused in periods of locomotion or around the start of locomotion? Would that increase the chances to capture hub-like dynamics in more cells? Anyway, given the small proportion (2/7) I would recommend authors to implement some form of surrogate analysis to discard chance results in fig.6i.*

First of all, we have slightly changed the method to determine statistical significance of ebGABAs as a population. We believe that the current method is more correct and robust. Like in the previous version of the manuscript, we sampled randomly picked GFP-negative cells to match the amount of ebGABAs in each FOV. But now we have repeated this procedure 1000 times to create a surrogate distribution. The proportion of modulated ebGABAs is considered significantly higher than the proportion of control cells if the former falls above the 95^th^ percentile of the surrogate distribution. The same procedure was employed to test for significance for the output functional connections and the proportion of hub cells. This method confirmed the significant effect of ebGABAs in terms of locomotion, assembly activity and combined locomotion-network effects. In addition, it revealed a difference close to significance (P = 0.05) for SCE modulation (see Figure 6 and Supplementary Figure 8).

With this method, we confirmed that ebGABAs display significantly higher output functional connectivity than control cells (P < 0.05, Figure 6k). This method allowed us to test against a null hypothesis for the proportion of hub cells. We found that the proportion of ebGABAs acting as hubs was higher than the proportion of control cells acting as hubs and this difference was at the edge of significance (P = 0.05).

We then followed the Reviewer’s suggestion and analyzed functional connectivity during periods of locomotion or rest (we did not restrict to the onset of locomotion because this gave too few data points to perform statistics). We found that ebGABAs showed significantly higher functional connectivity during locomotion (P < 0.05) but not rest (Supplementary Figure 8f), suggesting that locomotion periods drive the difference observed in the whole recordings.

*6- Fig.6k, It is unclear what the different shapes of the violin like plots represent. Please help the reader to understand the plot.*

We have added in the legends that “Dashed lines in violin plots show medians and quartiles.”

Please note that we have also increased the number of mice from which we quantified the expression of COUP-TFII in ebGABAs (reaching 5 mice). Additional brains confirmed the previous results, showing that very few ebGABAs (7 ± 2%) express this marker (page 14, line 228 and Supplementary Figure 3i).

**Reviewer comments, third version:**

Reviewer #2 (Remarks to the Author):

Authors have addressed the remaining comments satisfactorily. They have improved network analysis in Fig.6, which reads more robust. They also increased the number of mice used for histological quantification. I found the ms suitable for publication. Great work!

**Author rebuttal, third version:**
